# Development of highly efficient and specific base editors in *Actinobacillus succinogenes* for enhancing succinic acid production

**DOI:** 10.1186/s13068-023-02443-8

**Published:** 2023-12-12

**Authors:** Chunmei Chen, Pu Zheng, Pengcheng Chen, Dan Wu

**Affiliations:** The Key Laboratory of Industrial Biotechnology, Ministry of Education, School of Biotechnology, Jiangnan University, Wuxi, 214122 China

**Keywords:** Base editor, Cas9 nickase, Deaminase, Succinic acid, *Actinobacillus succinogenes*

## Abstract

The production of bio-succinic acid (SA) from renewable feedstocks is a promising and sustainable approach to mitigating the high carbon emissions associated with the current energy crisis. *Actinobacillus succinogenes* was recognized as one of the most promising SA producers; however, lack of genetic background and the scarcity of genetic manipulation tools hinder the improvement in *A. succinogenes* by metabolic engineering. Here, for the first time, we successfully developed a series of *A. succinogenes* base editors (BEs) mediated by the fusion of Cas9 nickase and deaminase, including CBE, ABE, Td-GABE, and Td-CBE. Among these, ABE and Td-CBE based on a fusion of Cas9 nickase and TadA-8e variant (*Escherichia coli* TadA) can efficiently convert A to G and C to T, respectively, with editing efficiencies of up to 100%. We also investigated the multiplex base editing of ABE and Td-CBE, and the results showed that the editing efficiency of ABE reached 100% for six sites and 10% editing efficiency of Td-CBE for two sites. In addition, cytosine base editors were applied to inactivate hypothetical sugar and SA transporters of *A. succinogenes*. We found that the inactivation of *Asuc_0914* encoding sucrose-specific IIBC subunit enhanced SA production, while the inactivation of hypothetical SA transporters *Asuc_0715* and *Asuc_0716* significantly reduced SA production. Therefore, the tools have great application potential in the metabolic engineering of *A. succinogenes*.

**Supplementary Information:**

The online version contains supplementary material available at 10.1186/s13068-023-02443-8.

## Introduction

Succinic acid (SA) is a crucial platform C4 chemical, since it has great commercial value and a wide range of application in chemical, food, pharmaceutical, and other fields of industry [1–3]. It is a precursor for the synthesis of high-value chemicals, such as adipic acid, tetrahydrofuran, and 1,4-butanediol [4, 5]. The demand for SA is expected to increase from 50,276 tons in 2017 to 97,099 tons in 2024 [4]. In addition, the global SA market is expected to grow at a compound growth rate, with the annual growth rate being approximately 27.4% and reaching approximately $1.8 billion by 2025 [6]. Bio-based SA production has attracted much attention because of its economical and great potential for future sustainable development. Various bacteria accumulate SA as a product. *A. succinogenes* is a strain that naturally accumulates high concentrations of SA and is recognized as one of the most promising SA producers [1, 7–11]. In microbial cell factories, advances in data using computational tools in omics and synthetic biology have made it possible to begin improving bacterial performance as biological production platforms. However, lack of genetic background and the scarcity of tools for genetic manipulation have led to further improvements in SA production of *A. succinogenes* to be behind those of other strains, such as *Escherichia coli*, *Corynebacterium glutamicum*, and *Mannheimia succiniciproducens* [2, 7, 12]. Although there were several gene knockout methods based on homologous recombination-mediated chromosomal integration and gene disruption [13–16], mutation efficiency in *A. succinogenes* was dissatisfactory, and the screening process was time-consuming and complicated. Nevertheless, the “scar” or screening marker usually remained in cells, preventing further engineered strains from being achieved.

Clustered regularly interspersed short palindromic repeats (CRISPR) and associated proteins (Cas) genome editing system was successfully employed in various plants, animals, and microorganisms [17–20]. The type II and type V CRISPR/Cas system was mostly used and developed because of its simple work element. Their signature genes were *cas9* and *cas12a*, respectively [21, 22]. In the CRISPR/Cas genome editing system, the complex of Cas protein and guide RNA (gRNA) can recognize the target sequence containing a specific protospacer‐adjacent motif (PAM) located immediately downstream of the protospacer, and then induces double‐strand breaks (DSB) [18, 23]. Depending on the organism, induced DSB can be repaired by either non-homologous end joining (NHEJ) or homology-directed repair (HDR) mechanisms; this enables gene knockout or targeted transgene knockin [24, 25]. However, DSB is lethal because of the weak repairability of many microorganisms; this limited the application of the system in many organisms [26, 27]. Recently, Cas nuclease and deaminase fusion has emerged as a new approach to genome editing that enables the direct, irreversible conversion of one target-DNA base into another in a programmable manner, without the collateral generation of undesirable DSBs [28–32]. It was successfully applied to humans, mice, rice, *C. glutamicum*, *Bacillus amyloliquefaciens*, *Staphylococcus aureus*, *cyanobacteria*, and *Bacillus subtilis* [26, 28, 33–36].

**Fig. 1:**
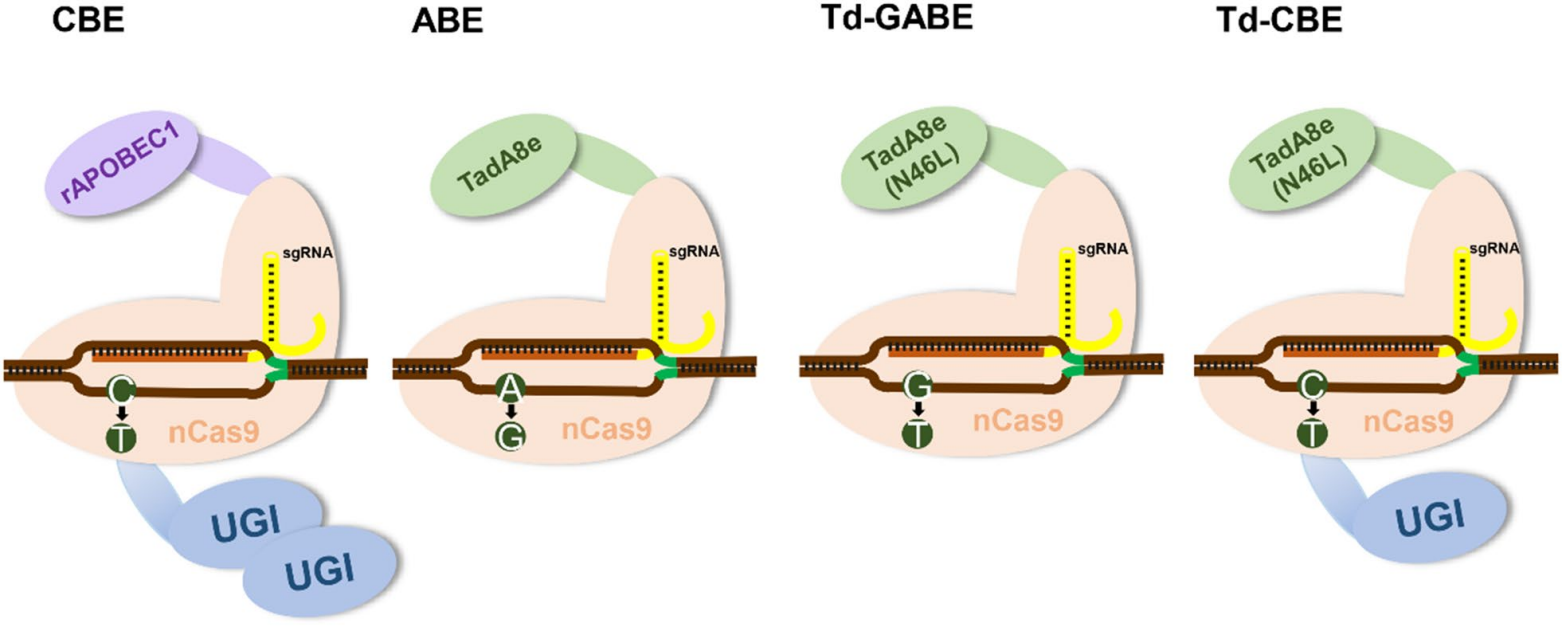
Schematic representation of the constructed four base editors’ complex form

In this study, base editors of *A. succinogenes* were developed for the first time. Four types of BEs for converting targeted C to T, A to G, and G to T in *A. succinogenes* were designed by engineering the fusion of Cas9 nickase and deaminase (Fig. 1). At the same time, their editing windows were identified, and their editing efficiencies were optimized, as well as their abilities for multiplex base editing. Gene silencing and single-base mutation in the genome of *A. succinogenes* were achieved through these base editors to explore its role in succinic acid synthesis, such as putative genes for associated transporters of sugars and SA. Here, inactivation of *Asuc_0914*, encoding of sucrose-specific IIBC subunit that may be involved in glucose transport, and inactivation of hypothetical SA transporters *Asuc_0715* and *Asuc_0716* were found to be associated with SA production. Thus, the tool allows for gene inactivation and point mutations in the genomes and may dramatically accelerate the exploration of *A. succinogenes* physiology and metabolic engineering.

## Results

### Design of CBE can convert targeted C to T in *A. succinogenes*

**Fig. 2:**
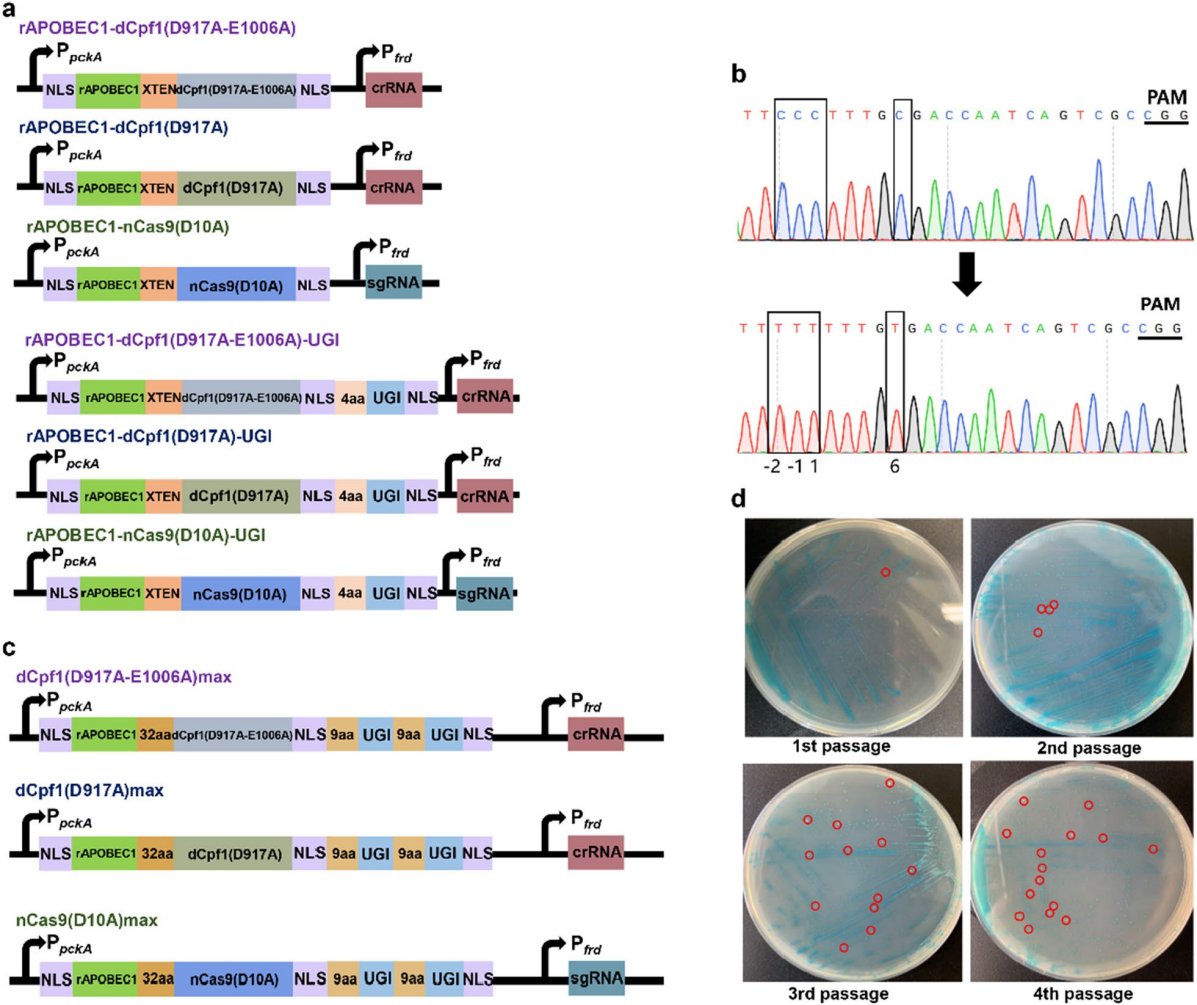
The engineered base-editing system enables C-to-T conversion in *A. succinogenes*. **a** Architectures of CBEs. rAPOBEC1-nCas9(D10A), rAPOBEC1-dCpf1(D917A), and rAPOBEC1-dCpf1(D917A-E1006A), a fusion protein composed of a deaminase rAPOBEC1 at the N terminus, and nCas9(D10A)/dCpf1(D917A)/dCpf1(D917A-E1006A) at the C terminus; rAPOBEC1-nCas9(D10A)-UGI/rAPOBEC1-dCpf1(D917A)-UGI/rAPOBEC1-dCpf1(D917A-E1006A)-UGI, a fusion protein composed of a deaminase rAPOBEC1 at the N terminus, nCas9(D10A)/dCpf1(D917A)/dCpf1(D917A-E1006A) at the middle, and UGI at the C terminus. **b** The rAPOBEC1-nCas9(D10A)-UGI enables C-to-T base-editing targeting lacZ in *A. succinogenes*. **c** Architectures of optimized CBEs. **d** The subculture improves gene editing efficiency (the strains were streaked on TSB-X-gal plate). The red dots were the marked white colonies

In our previous study, the *cas9* from *S. pyogenes* and *cpf1* from *Francisella novicida* were successfully expressed in *A. succinogenes* [37]. To construct the base editor for *A. succinogenes*, cytidine deaminase rAPOBEC1 from rat was fused with the amino terminus of dCpf1(D917A-E1006A), dCpf1(D917A), and nCas9(D10A) (Fig. 2a). The plasmid pLGZ922 was selected to drive the editing element, and the *frd* promoter was used to drive the gRNA element [37–39]. To facilitate rapid phenotype identification, *lacZ* gene (*Asuc_1398*), which encodes a β-galactosidase in *A. succinogenes*, was selected as the editing target. The mutant will not show blue phenotype on the TSB plates containing X-gal by disrupting *lacZ* [13].

The relevant gRNAs were designed to generate a C-to-T substitution and introduced a STOP codon to break the *lacZ* gene (Additional file 1: Table S3). After transformation by electroporation, no white colony was obtained on the TSB plate containing 100 μg/mL X-gal, and ten blue colonies were randomly picked and sequenced. Similarly, no editing mutants were obtained. The DNA repair of cells in response to U:G heteroduplex DNA may be the cause of the base-editing failure. Uracil DNA glycosylase (UDG) catalyzes the removal of U from cellular DNA and initiates base excision repair, with reducing U:G pairs to C:G pairs being the most common result. Uracil DNA glycosylase inhibitor (UGI) from *Bacillus subtilis* bacteriophage PBS1 hinders UDG activity [28, 40]. Therefore, fusing the UGI to the carboxy-terminus of dCpf1(D917A-E1006A), dCpf1(D917A), and nCas9(D10A) improves the editing efficiency for generate DNA base editors (Fig. 2a). After transformation of *A. succinogenes*, no white colonies were obtained. Multiple subcultures can improve the editing efficiency in some studies [41]. However, no white colony was obtained after transformation of rAPOBEC1-dCpf1(D917A-E1006A)-UGI and rAPOBEC1-dCpf1(D917A)-UGI to *A. succinogenes* and culture for first and second passages. Simultaneously, ten blue colonies were randomly picked and sequenced. The sequencing results showed that no colony had base mutation, indicating that rAPOBEC1-dCpf1(D917A-E1006A)-UGI and rAPOBEC1-dCpf1(D917A)-UGI had no base-editing ability in *A. succinogenes*. Only one white colony was obtained by transforming rAPOBEC1-nCas9(D10A)-UGI and culturing for the first and second passages. One white colony and nine random blue colonies from the first and second passages were picked and sequenced. The sequencing results showed that only two white colonies had base mutation at Cs positions of -2, -1, 1, and 6 upstream of CGG PAM, which indicated that rAPOBEC1-nCas9(D10A)-UGI had the potential for base editing in *A. succinogenes* (Fig. 2b).

In addition, glycosylase base editors (GBEs) consist of a Cas9 nickase, a cytidine deaminase and a UDG, cause C-to-A transversions in *Escherichia coli* and C-to-G transversions in mammalian cells [42]. To construct GBE, *udg* from *E. coli* was fused to C-terminal of rAPOBEC1-nCas9(D10A), but no C-to-G or C-to-A conversion was observed, indicating that rAPOBEC1-nCas9(D10A)-udg could not convert C-to-G or C-to-A in *A. succinogenes*.

### Optimization of CBE and identification of editing window in *A. succinogenes*

Although rAPOBEC1-nCas9(D10A)-UGI could convert C to T successfully in *A. succinogenes*, the efficiency was unsatisfactory. The number of cells expressing base editors and/or functional editor proteins produced by each cell is a major bottleneck of editing efficiency [30]. In addition, extending the rAPOBEC1-nCas9(D10A) linker and nCas9(D10A)-UGI linker can offer higher efficiencies of C-to-T conversion, higher product purities, and lower indel rate [43]. To improve the editing efficiency, extending the rAPOBEC1-nCas9(D10A) linker to 32aa amino acids and nCas9(D10A)-UGI linker to 9aa amino acids, and another UGI was connected with the carboxy-terminus of UGI by the linker of a 9aa amino acid to yield nCas9(D10A)max. In addition, to determine whether the BE based on dCpf1 cannot be edited in *A. succinogenes*, the linker length between fusion proteins and UGI copy number were also increased, yielding dCpf1(D917A-E1006A)max, and dCpf1(D917A)max (Fig. 2c). After transformation, only nCas9(D10A)max achieved white colony after first passage, suggesting that nCas9(D10A)max could give rise to mutation in *A. succinogenes*. The efficiency of nCas9(D10A)max was detected after multiple subcultures on TSB plates containing 100 μg/mL X-gal. As shown in Fig. 2d, the number of white colonies increased with the number of passages, suggesting that editing efficiencies were increased by multiple subcultures and that the number of fourth passage was similar to the number of third passage. The third passage was used in all subsequent studies.

**Fig. 3:**
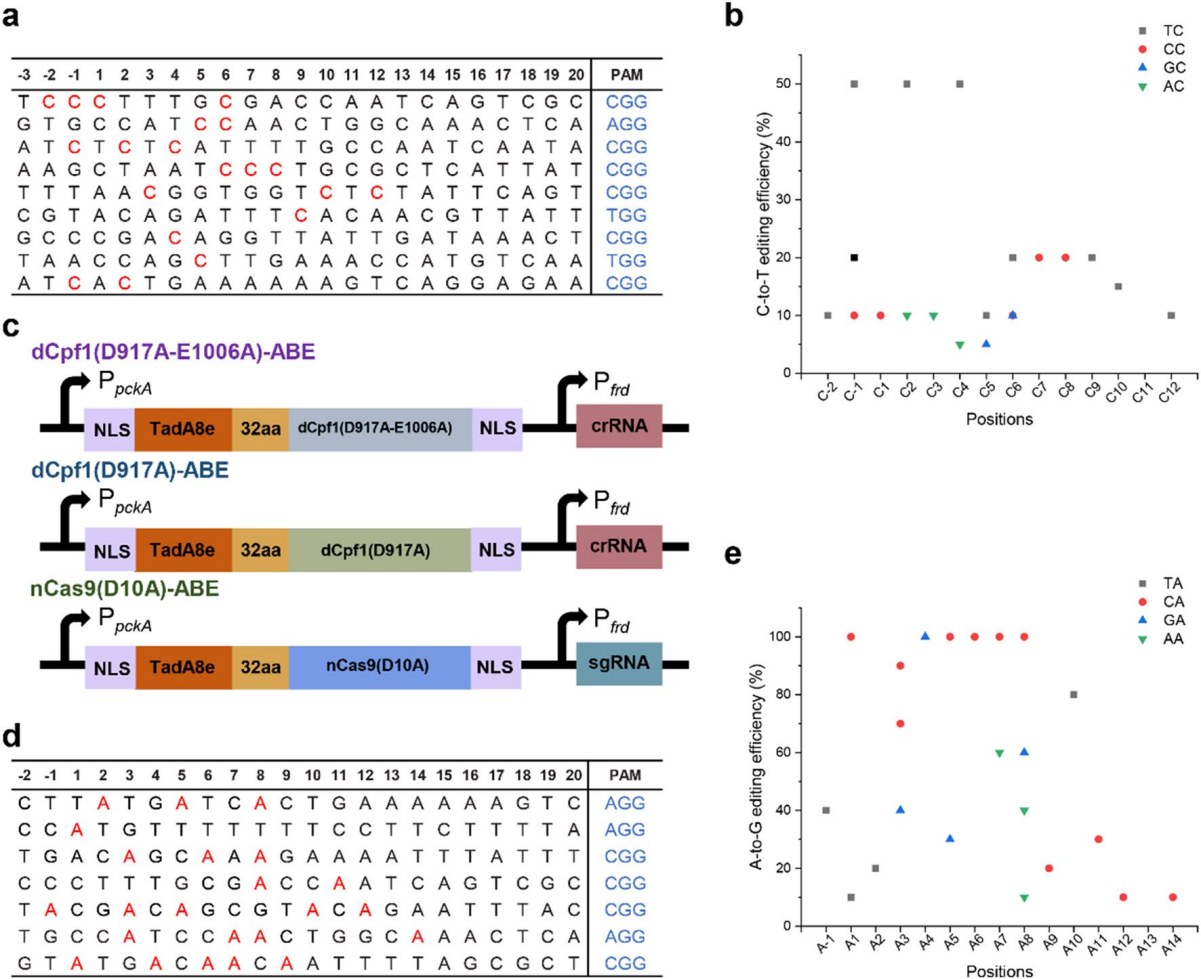
The window and efficiency of the optimized base-editing system enables C-to-T / A-to-G conversion. **a** The editing region of the targeting gene by the optimized base-editing system. The red letters were the positions of successful editing. **b** Editing efficiency of the optimized base-editing system enables C-to-T conversion by different sgRNAs in *A. succinogenes*. The graph shows the percentage of total DNA sequencing reads containing T at each of the successful editing target C positions by the optimized CBE. **c** Architectures of the ABEs. **d** The editing region of the targeting gene by the ABE. The red letters were the positions of successful editing. **e** Editing efficiency of the base-editing system enables A-to-G conversion by different sgRNAs in *A. succinogenes*. The graph shows the percentage of total DNA sequencing reads containing G at each of the successful editing target A positions by ABE

To evaluate the availability of nCas9(D10A)max, other two sgRNAs were designed and employed for modifying *lacZ* gene in *A. succinogenes *(Additional file 1:Table S3). The sequencing results showed that the mutants were obtained by only one sgRNA, suggesting that the availability of nCas9(D10A)max was affected by the position of the editing target C. In addition, the analysis found that the preference of editing was TC (the second nucleotide C was the target); this was consistent with the previous studies [28]. To identify the efficiency and window of base editing, nine sgRNAs were designed and employed for other positions (Fig. 3a). Sequencing results showed that C-to-T conversion was achieved at Cs position of –2 to 12 except for position 11, and that the efficiency of editing was 5%–50% (Fig. 3b). These results suggest that the editing window of nCas9(D10A)max was 14 nucleotides and preferentially edits the motif TC.

Anc689, optimization, and ancestral reconstruction containing 36 amino acid substitutions relative to rAPOBEC1 replaced rAPOBEC1 of nCas9(D10A)max to construct Anc689-nCas9(D10A)max to improve expected editing efficiency. The results showed that the conversion of C to T at Cs positions of 6, 7, and 8 were achieved and that the editing efficiency was 20%–30% (Additional file 1: Figure S1), indicating that the efficiency of Anc689-nCas9(D10A)max was similar to that of nCas9(D10A)max.

### Design of ABE can convert targeted A to G in *A. succinogenes*

On the basis of a series of CBEs which were constructed in this study, we fused the TadA-8e variant (derived from *E. coli* TadA) amino terminus of dCpf1(D917A-E1006A), dCpf1(D917A), and nCas9(D10A) to generate the ABEs (Fig. 3c). After transformation by electroporation, ten colonies were randomly picked for sequencing. The results showed that A-to-G point mutation was obtained by only nCas9(D10A)-ABE and that the editing efficiency was 100%, whereas no A to G point mutation was detectable by dCpf1(D917A-E1006A)-ABE and dCpf1(D917A) under similar conditions. These results were consistent with the above CBEs, showing that FnCpf1 did not work successfully with deaminase in *A. succinogenes*. The selection of suitable Cpf1 nickase or other Cpf1 might be a further direction for developing BEs in *A. succinogenes*.

The editing window of TadA-8e for SpCas9 was positions 4–8 [31]. To further determine the editing window and the editing efficiency of nCas9(D10A)-ABE, several endogenous genomic sites were designed (Fig. 3d). The sequencing results showed that the A-to-G editing mutation was obtained at all target sites and that the A-to-G editing efficiency was 100%, with maximum editing efficiency achieved at positions 4–8 (Fig. 3e). In addition, nCas9(D10A)-ABE exhibited broad editing window from positions—1 to 14 within the protospacer, thus providing more mutation-site selection for *A. succinogenes*.

### Design of TadA8e-derived base editor can convert targeted G to T or C to T in *A. succinogenes*

**Fig. 4:**
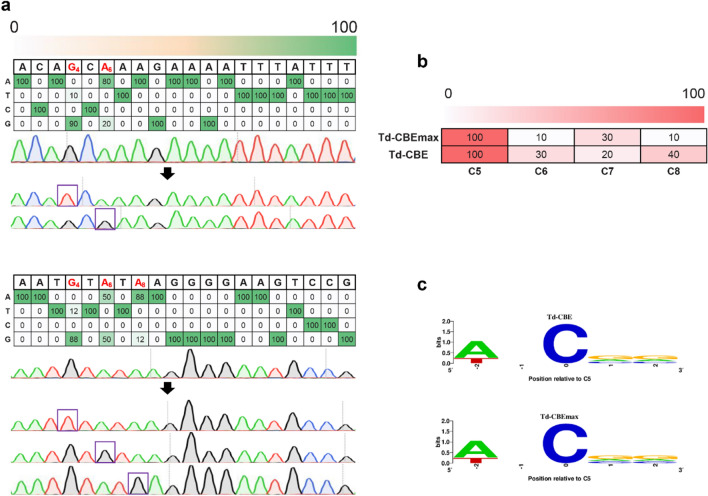
The editing efficiencies and motif preference of BEs. **a**Td-GABE mediated mutation in *A. succinogenes*. The sequence of protospacer is shown at the top. Underneath each sequence are the percentages of total sequencing reads with the corresponding base. **b** Heatmaps showing the on-target C-to-T editing efficiencies of Td-CBE and Td-CBEmax in *A. succinogenes*. **c** Motif visualization of the indicated Td-CBEs using sgRNAs with cytosine at position 5 of the protospacer

Although CBEs were successfully developed in the above works, the editing efficiency was dissatisfactory. Recent studies have shown that the TadA8e variant (with an N46L mutation) eliminates adenine deaminase activity, resulting in a TadA8e-derived C-to-G base editing (Td-CGBE) and further fusion with UGI resulting in C-to-T base editing (Td-CBE) that can be edited efficiently and precisely [44]. The mutation point of N46L was introduced into nCas9(D10A)-ABE to generate Td-CGBE, which resulted in C-to-G base editing. However, it was different from their studies in which the A-to-G base-editing efficiency of Td-CGBE remained 20%, and no C-to-G base editing was detectable in *A. succinogenes* (Fig. 4a). Surprisingly, G-to-T base editing was detected at position G4. To confirm the G-to-T editing capability of Td-CGBE at position G4, the other three sgRNA of AG_4_*N, CG_4_*N, and TG_4_*N motifs were designed. Td-CBE induced G-to-T conversion at position G4 with only TG_4_*N motif (12%), and A-to-G conversion at position 6 (50%) and position 8 (12%) (Fig. 4a), suggesting that TadA8e-N46L was capable of G-to-T conversion and A-to-G conversion and was efficient on GG_4_*N and TG_4_*N motifs in *A. succinogenes*. We renamed it TadA8e-derived G-to-T and A-to-G base editor (Td-GABE).

Fusion with one copy of UGI and two copies of UGI to generate Td-CBE and Td-CBEmax was carried out. The sequencing results showed that Td-CBE and Td-CBEmax efficiently induced C-to-T conversion at position C5 with efficiency of up to 100%, suggesting that they were highly efficient (Fig. 4b). The editing window of Td-CBE was positions 6–8 [44]. To investigate the editing window and efficiency of Td-CBE and Td-CBEmax in *A. succinogenes*, several targets were tested. It was found that Td-CBE and Td-CBEmax showed efficient C-to-T editing at positions 6–8, similar to a previous study [44] (mean efficiency of 10%–40% for Td-CBE and Td-CBEmax). In addition, similar to eTd-CBEmax [44], efficient C-to-T editing at position C5 (100%) was observed. The motif preferences of Td-CBE and Td-CBEmax were detected at position C5. The results showed that Td-CBE and Td-CBEmax had no strict sequence context requirement at position 5 of protospacers, suggesting that Td-CBE and Td-CBEmax provided efficient and precise editing tools for single nucleotides in *A. succinogenes* (Fig. 4c).

### Multiple targets simultaneously convert A to G or C to T in *A. succinogenes*

Simultaneous action of multiple sites can save a large amount of time and processes, providing great convenience for metabolic engineering. However, this was not feasible in the past in *A. succinogenes* because of lack of a CRISPR/Cas genome editing system. In this study, several base editors were first developed and represented excellent performance in *A. succinogenes*. Because nCas9(D10A)-ABE and Td-CBEs showed highly efficient base editing in *A. succinogenes*, we selected them for the subsequent experiment.

**Fig. 5:**
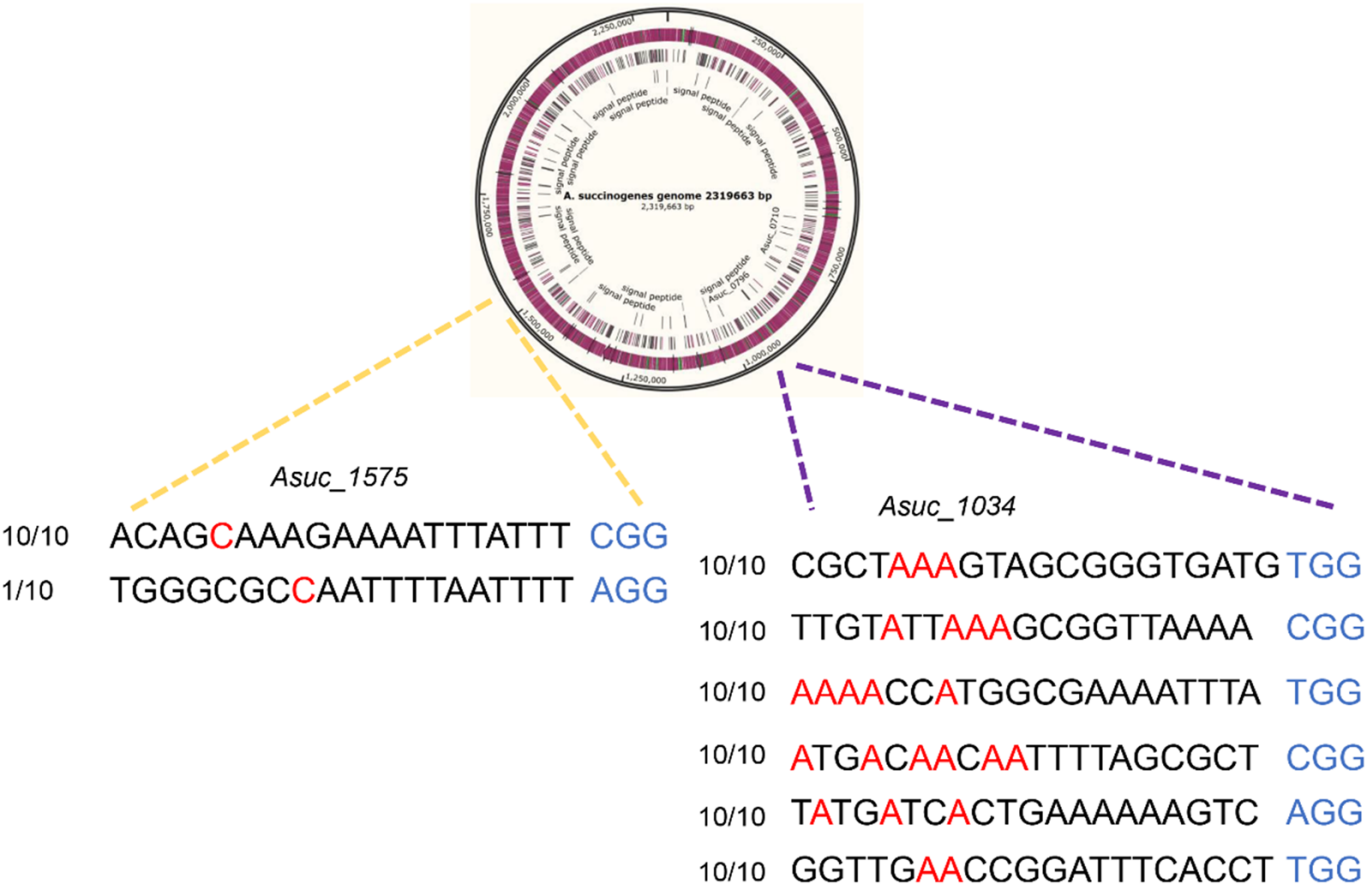
Multiplexed cytosine base editing and adenine base editing using nCas9(D10A)-ABE and Td-CBE, respectively. The red bases were possible editing targets

To confirm the possibility of multiple targets being edited simultaneously in *A. succinogenes*, nCas9(D10A)-ABE and Td-CBEs were constructed containing two sgRNAs. *Asuc_1034* and *Asuc_1575* were target genes of nCas9(D10A)-ABE and Td-CBEs, respectively. After being transformed to *A. succinogenes*, ten colonies were randomly picked for sequencing. The results showed that simultaneous A-to-G base editing at two sites targeting *Asuc_1034* was achieved with efficiency of 100% by nCas9(D10A)-ABE, suggesting that multiple base editing by nCas9(D10A)-ABE was feasible (Fig. 5). Thereafter, we constructed sgRNA arrays by assembly of three sgRNAs, four sgRNAs, five sgRNAs, and six sgRNAs used for *Asuc_1034*, and inserted them into nCas9(D10A)-ABE, resulting in multiple loci editing plasmids. The results showed that the A-to-G editing efficiency by nCas9(D10A)-ABE were 100% for each site, indicating that nCas9(D10A)-ABE exhibited efficient A-to-G conversion at multiple loci in *A. succinogenes*. However, simultaneous C-to-T base editing at two sites of *Asuc_1575* was achieved with efficiency of 10% by Td-CBE and Td-CBEmax. The editing efficiency of the first site was 100%, and the editing efficiency of the second site was only 10%. These results demonstrated that nCas9(D10A)-ABE, Td-CBE, and Td-CBEmax can perform multiple base editing, but nCas9(D10A)-ABE is more efficient.

### Application of BEs in *A. succinogenes* for SA production

**Fig. 6:**
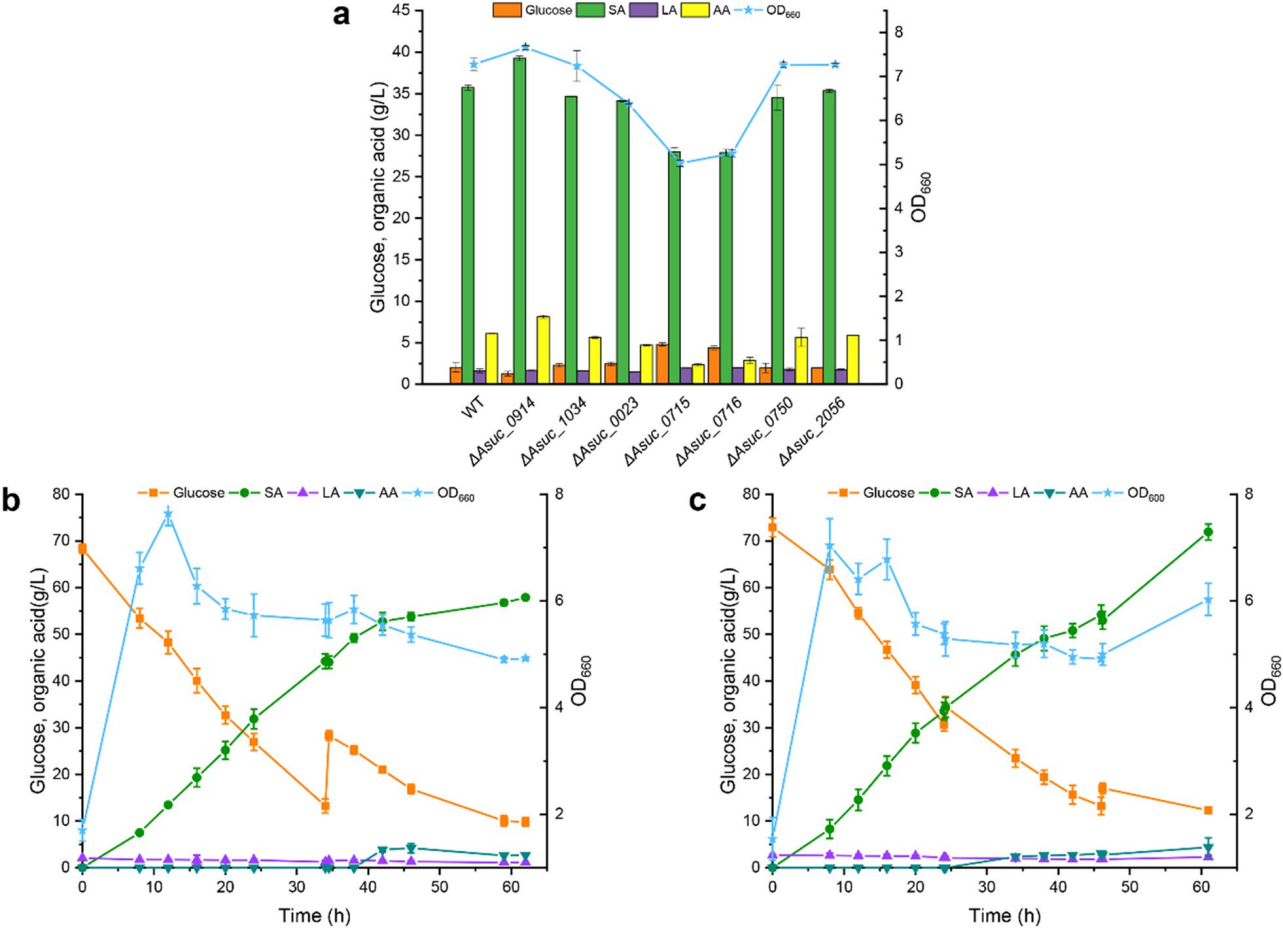
Evaluation of different fermentation and metabolic parameters in wild-type *A. succinogenes* and mutants. **a** Evaluation of glucose and organic acid in wild-type *A. succinogenes* and mutants by shake-flask fermentation. **b** Evaluation of glucose, organic acid, and cell growth in the wild type by a 3 L scale fed-batch fermentation. **c** Evaluation of glucose, organic acid, and cell growth in ΔAsuc_0914 by a 3 L scale fed-batch fermentation. All data were the average of three independent experiments

To illustrate the wide application of the BEs in *A. succinogenes*, we used them for metabolic engineering with a view of enhancing the production of SA. Transporters of substrates and products were crucial for the production of SA in cells. The *SucE1* gene of *C. glutamicum* and *yjjPB* gene of *E. coli* were identified as succinate exporters that were required for succinate export [45, 46]. Two JEN family carboxylate transporters (PkJEN2-1 and PkJEN2-2) of *Pichia* *kudriavzevii* were found to effectively import succinate. Simultaneous inactivation of both PkJEN transporters enhanced extracellular accumulation of SA in the late stage of fermentation [47]. On the other hand, inactivation of *ptsG*, which encodes glucose-specific permease of the phosphotransferase system–protein EIICB^glc^, shifted the fermentative metabolism strongly toward succinate in fermentation of glucose by *E. coli* [48]. In addition, *mgtA* and *mgtBC* genes were found to be magnesium transporters in *E. coli* and *Salmonella enterica* serovar Typhimurium [49, 50]. However, transporters of sugars and SA remained poorly understood in *A. succinogenes*. By BLAST, *Asuc_0023* and *Asuc_0715-0716* were found to be homologous with succinate exporter, and *Asuc_0750* and *Asuc_2056* were found to be homologous with succinate importer in *A. succinogenes*. *Asuc_0914* was homologous with *ptsG*, and *Asuc_1034* was homologous with a magnesium transporter. A stop codon was expectedly created in *A. succinogenes* using nCas9(D10A)-CBEmax or Td-CBE to target the above genes (Additional file 1: Figure S2). The SA production of wild type and mutant were determined by shake-flask fermentation containing 50 g/L glucose. As shown in Fig. 6a, the SA productions of Δ*Asuc_0023*, Δ*Asuc_0750*, Δ*Asuc_2056*, and Δ*Asuc_1034* were similar to those of the wild type. Interestingly, Δ*Asuc_0914* displayed efficient glucose utilization and an increase in SA production, which was in agreement with the results of *ptsG* inactivation in *E. coli*, resulting in SA production of approximately 39.26 g/L. In addition, the SA production of Δ*Asuc_0715* and Δ*Asuc_0716*, as well as their OD_660_ values, were significantly lower, indicating that inactivation of *Asuc_0715* and *Asuc_0716* may affect the transport of SA, resulting in intracellular SA accumulation, which was not conducive to growth and acid production.

The wild-type strain and Δ*Asuc_0914* strain were used to perform fermentation in a 3 L fermenter. Time courses of SA production and other parameters were measured (Fig. 6b and c). We obtained 71.92 g/L SA with a yield of 1.03 g/g at 60 h of fermentation. Compared with the original strain, the titer of SA and the yield increased 1.24-fold, suggesting that deletion of *Asuc_0914* may alter the uptake of sugar and maintain cell activity, resulting in more SA production.

## Discussion

*A. succinogenes* was a promising native succinic acid producer owning to its broad utilization spectrum of carbohydrates, and high tolerance under osmotic pressure and high concentrations’ succinate [51, 52]. However, the development of genome editing tools for *A. succinogenes,* especially mediated by CRISPR/Cas system, lagged behind other bacteria. There were three main reasons that hindered development and application of CRISPR/Cas genome editing system in *A. succinogenes*: (1) the lack of expressing plasmids, (2) no promoters to express gRNA element, and (3) weak DNA repair ability. To develop a CRISPR/Cas genome editing system, the one and only plasmid pLGZ922 was chosen as backbone, which has been demonstrated to express foreign proteins [53]. The endogenous *frd* (fumarate reductase) promoter was used to drive gRNA element. However, no positive colony was observed due to its weak DNA repair ability. Recently, although we expressed Cas9 from *Streptococcus pyogenes* and Cpf1 from *Francisella tularensis* and developed a CRISPR interference (CRISPRi)-mediated gene repression system based on dCpf1 in *A. succinogenes* [37], the existence of plasmid limits its wide application. In this study, we developed a series of BEs by fusing Cas protein and deaminase to convert C-to-T, A-to-G, G-to-T in *A. succinogenes*. Unlike CRISPRi, however, we found that the dFnCpf1 was incompatible with rAPOBEC1/TadA8e, while nCas9(D10A) with TadA8e exhibit excellent performance for base editing in *A. succinogenes*. Thus, we successfully achieved precise genome editing at a single-nucleotide resolution with Td-CBE and further multiplex genome editing with ABE and Td-CBE, which not only provided a new genome editing tools, but also laid the foundation for the development of CRISPR/Cas genome editing system in *A. succinogenes*. Base editor perform genome editing without DSB, it is not fully applicable to introduce STOP codon in the open-reading frames of *A. succinogenes* due to the PAM dependence of current Cas9-guided base editor. Therefore, an efficient base-editing toolbox of debottlenecking PAM is further required for *A. succinogenes*. Different Cas proteins such as Cpf1 that requires a T-rich PAM sequence for target-DNA recognition were employed to develop base editor by fusing deaminase with UGI, yielding C-to-T conversion in human cells [32, 54]. However, dFnCpf1-BEs did not shown detectable base editing in this study, indicating that FnCpf1 was not compatible to rAPOBEC1 with UGI in *A. succinogenes*. Currently, highly efficient PAM-less base-editing toolbox for *B*. *subtilis* based on a variant of Cas9 nickase (nSpRY) was developed, yielding PAM-less adenine and/or cytosine base editors [55]. Therefore, it is expected that replacing Cas variants would further improve the applicability of BEs.

*A. succinogenes* produces SA as part of a mixed acid fermentation, along with the production of acetate and formate as by-products, leading to difficult separation and purification of SA and increases the production costs. It is desirable to genetically engineer *A. succinogenes* to produce SA as the sole fermentation end product. Carbon flux distribution to succinate and alternative products was determined for understanding of the metabolic pathways by gas chromatography–mass spectrometry and nuclear magnetic resonance spectroscopy, and found that formate and pyruvate dehydrogenases were contributing to NADH production, and oxaloacetate and malate were being decarboxylated to pyruvate [56, 57]. Further, implementation of metabolic engineering strategies for interruption of the competing pathways and enhanced biosynthetic pathway flux still did not achieve desired SA yields [14], suggesting the existence of significant opportunity for additional strain engineering. Transporters of substrates and products were crucial for the target product in cell factory. Sugar and SA transporters of microorganisms commonly used in the synthesis of SA, such as *E. coli*, *C. glutamicum*, and *P. kudriavzevii*, were identified [45–48]. However, no genes were identified to encode sugar and SA transporters in *A. succinogenes*. This study focused on the sugar and SA transporters and found that several hypothetical transporters were required for SA synthesis in *A. succinogenes*, providing the alternative promising metabolic engineering strategies for SA production.

## Conclusions

In summary, we have developed a series of BEs by fusion of Cas nuclease and cytidine/adenine deaminase in *A. succinogenes* for the first time. Here, two base editors, ABE and Td-CBE, were engineered with Cas9 and TadA mutant, which can efficiently convert A to G and C to T, respectively, with editing efficiency of up to 100%. Further, multiplex genome editing was achieved by these two BEs. We applied the BEs to inactivation of sugars and SA transporters to study their effects on SA production in *A. succinogenes*, and we found that *Asuc*_0914, *Asuc*_0715, and *Asuc*_0716 are significantly associated with SA production. This study established an efficient and fast genetic manipulation tool, which will provide critical insights into the development of a CRISPR/Cas genome editing system and SA production through metabolic engineering of *A. succinogenes*.

## Materials and methods

### Strains, primers, plasmids, sgRNAs, media, and culture condition

The strains and plasmids used in this study are listed in Additional file 1: Table S1, and the primers are listed in Additional file 1: Table S2. *E. coli* JM109 was used as the host for the plasmid clone and cultured with LB medium at 37 °C or on LB agar plate with ampicillin (100 μg/mL) when necessary. *A. succinogenes* CGMCC1593 was anaerobically cultured with TSB medium or TSB agar plate with ampicillin (100 μg/mL) or X-gal when necessary. The fermentation medium was described in Reference [37]. The sgRNAs were designed online (https://chopchop.cbu.cib.no/), and the protospacer sequences are listed in Additional file 1: Table S3.

### Plasmid construction

The plasmids pLGZ-nCas9(D10A), pLGZ-dCpf1(D917A), and pLGZ-dCpf1(d917A-E1006A), reported in our previous study, harbor Cas protein and sgRNA/crRNA. They were used as the backbone to construct base editors [37]. Condon-optimized rAPOBEC1, UGI, and TadA8e were synthesized by GENEWIZ.

To construct a cytosine base editor based on nCas9(D10A) or dCpf1, rAPOBEC1 was fused with the amino terminus of dCpf1(D917A-E1006A), dCpf1(D917A), and nCas9(D10A). Here, the plasmids pLGZ-nCas9(D10A), pLGZ-dCpf1(D917A), and pLGZ-dCpf1(D917A-E1006A), respectively, were used as the template to generate linearized vectors for constructing different CBEs. The linearized vectors and rAPOBEC1 fragment were amplified using the relevant primers. The PCR product of rAPOBEC1 fragment was assembled into linearized vectors using ClonExpress II One Step Cloning Kit (Vazyme, China) to yield rAPOBEC1-nCas9(D10A), rAPOBEC1-dCpf1(D917A), and rAPOBEC1-dCpf1(D917A-E1006A), respectively (Fig. 1a). Thereafter, the linearized vectors were amplified using rAPOBEC1-nCas9(D10A), rAPOBEC1-dCpf1(D917A), and rAPOBEC1-dCpf1(D917A-E1006A) as the template by the relevant primers. The UGI fragment was amplified using the primers and assembled into the linearized vectors to yield rAPOBEC1-nCas9(D10A)-UGI, rAPOBEC1-dCpf1(D917A)-UGI, and rAPOBEC1-dCpf1(D917A-E1006A)-UGI, respectively. Similarly, another UGI was assembled into the linearized vector rAPOBEC1-nCas9(D10A)-UGI, rAPOBEC1-dCpf1(D917A)-UGI, and rAPOBEC1-dCpf1(D917A-E1006A)-UGI, to generate nCas9(D10A)-CBEmax, dCpf1(D917A)-CBEmax, and dCpf1(D917A-E1006A)-CBEmax, respectively.

Similarly, the plasmids pLGZ-nCas9(D10A), pLGZ-dCpf1(D917A), and pLGZ-dCpf1(D917A-E1006A) were used as the templates to generate linearized vectors for constructing different ABEs. The TadA8e fragment was amplified and assembled into the linearized vector to construct TadA8e-nCas9(D10A), TadA8e-dCpf1(D917A), and TadA8e-dCpf1(D917A-E1006A), respectively. Td-CGBE was created by PCR to introduce an N46L mutation. Thereafter, one copy of UGI and two copies of UGI were assembled into Td-CGBE to generate Td-CBE and Td-CBEmax, respectively.

To cure the plasmid, the temperature-sensitive replicon Rep was amplified from the plasmid pKCcas9dO and assembled into the plasmid of BE to yielding Rep-nCas9(D10A)-ABE, Rep-Td-CBE, and Rep-Td-CBEmax.

### Methods for the transformation and mutant modification of *A. succinogenes*

For *A. succinogenes*, the method for the transformation refers to references [14, 37].

After transformation, we randomly transferred one colony to TSB liquid medium containing 100 μg/mL ampicillin for cultivation for 24 h and then spread it on TSB agar plate overnight. Once the colonies had developed, the colony PCR was performed by randomly picking ten colonies, and the PCR products were sent for sequencing. The mutation was determined based on the sequencing results

$$\begin{gathered} {\text{Editing efficiency}} = \frac{{{\text{The number of mutated colonies}} }}{{\text{The total number of picked colonies}}} \times 100\% \quad {\text{or}}\quad \hfill \\ \frac{{{\text{The}}\;{\text{mutated}}\;{\text{number}}\;{\text{of}}\;{\text{target}}\;{\text{positions}}}}{{{\text{The}}\;{\text{total}}\;{\text{sequencing}}\;{\text{number}}\;{\text{of}}\;{\text{target}}\;{\text{positions}}}} \times 100\% . \hfill \\ \end{gathered}$$

### Plasmid curing

To curie plasmids, a temperature-sensitive replication protein, Rep from the plasmid pKCcas9dO, was introduced into the plasmid of BEs. After the mutant was obtained at 30 °C, the mutant was cultured at 37 °C for 24 h to eliminate the plasmid. The target mutants that did not grow in the presence of antibiotics and that grew in the absence of antibiotics were the target strains. However, *A. succinogenes* grows slowly at 30 °C, which leads to a prolonged experiment period. Indeed, the other plasmid curing procedure was performed by subculture of mutants in TSB medium overnight and plating on TSBG agar without antibiotics. Likewise, the target mutants that did not grow in the presence of antibiotics and that grew in the absence of antibiotics were the target strains.

### Construction of multiplex-site base editor

To construct multiplex-site ABE, one, two, three, four, five, and six sgRNAs were designed and assembled by ClonExpress II One Step Cloning Kit (Vazyme, China) by targeting different sites on *Asuc_1034*. These sgRNAs were driven by the *frd* promoter and assembled into the nCas9(D10A)-ABE plasmid. Similarly, two sgRNAs were assembled into Td-CBE and Td-CBEmax to target *Asuc_1575*.

### Fermentation and analytical methods

*A. succinogenes* was cultivated anaerobically in 25 mL of TSB medium at 37 °C for 12–16 h, and a 10% v/v inoculum was added into shake flasks containing 50 g/L glucose fermentation medium with the pH regulator MgCO_3_ powder for 48 h. For a 3 L fermenter, the temperature and agitation were 38 °C and 200 rpm, respectively. MgCO_3_ was used as pH regulator in the early stage of fermentation, and 3 M Na_2_CO_3_ was maintained at pH 6.5 in the late stage of fermentation. The initial glucose concentration was 70 g/L, and we supplemented glucose to maintain it between 15 and 30 g/L when the glucose fell to below 15 g/L.

The optical density of *A. succinogenes* was monitored by spectrophotometry at 660 nm (OD_660_). Glucose and organic acids in the fermentation broth were analyzed by HPLC. The special determination methods refer to Reference [37].

### Supplementary Information

**Additional file 1: Table S1.** Strains and plasmids used in this study. **Table S2.** The primer sequences used in this study. **Table S3.** The protospacer sequences used in this study. **Figure S1.** The sequencing results of Anc689-nCas9(D10A) targeting *LacZ *gene in *A. succinogenes*. **Figure S2.** The sequencing results of STOP codon introduction targeting genes of transporters in *A. succinogenes*. **Sequences S1.** The nCas9(D10A)-CBEmax sequences in this study. **Sequences S2.** The nCas9(D10A)-ABE sequences in this study. **Sequences S3.** The Td-GABE sequences in this study. **Sequences S4.** The Td-CBE sequences in this study.

## Data Availability

All data generated or analyzed during this study are included in this published article (and its additional files). All vectors generated in this study can be obtained from the corresponding author on reasonable request.
