# Supplementary material for: Development of highly efficient and specific base editors in Actinobacillus succinogenes for enhancing succinic acid production
Source: Biotechnol Biofuels Bioprod. 2023 Dec 12;16:192. doi: 10.1186/s13068-023-02443-8 (PMC10717943; doi:10.1186/s13068-023-02443-8)
Supplement: Supplementary file 1 — Additional file 1: Table S1. Strains and plasmids used in this study. Table S2. The primer sequences used in this study. Table S3. The protospacer sequences used in this study. Figure S1. The sequencing results of Anc689-nCas9(D10A) targeting LacZ gene in A. succinogenes. Figure S2. The sequencing results of STOP codon introduction targeting genes of transporters in A. succinogenes. Sequences S1. The nCas9(D10A)-CBEmax sequences in this study. Sequences S2. The nCas9(D10A)-ABE sequences in this study. Sequences S3. The Td-GABE sequences in this study. Sequences S4. The Td-CBE sequences in this study. [file 13068_2023_2443_MOESM1_ESM.docx]

**Title: Development of highly efficient and specific base editors in *Actinobacillus succinogenes* for enhancing succinic acid production**

**Authors:** Chunmei Chen, Pu Zheng^[[1]](#footnote-1)^*, Pengcheng Chen, Dan Wu

The Key Laboratory of Industrial Biotechnology, Ministry of Education, School of Biotechnology, Jiangnan University, Wuxi 214122, China.

*Corresponding author: Pu Zheng

Tel./ fax: +86 510 8591 8156.

E-mail address: zhengpu@jiangnan.edu.cn.

**Additional information**

**Additional Table S1** Strains and plasmids used in this study

**Additional Table S2** The primer sequences used in this study

**Additional Table S3** The protospacer sequences used in this study

**Additional Fig. S1** The sequencing results of Anc689-nCas9(D10A) targeting *LacZ* gene in *A. succinogenes*

**Additional Fig. S2** The sequencing results of STOP codon introduction targeting genes of transporters in *A. succinogenes*

**Additional sequences S1 The nCas9(D10A)-CBEmax sequences in this study**

**Additional sequences S2 The nCas9(D10A)-ABE sequences in this study**

**Additional sequences S3 The Td-GABE sequences in this study**

**Additional sequences S4 The Td-CBE sequences in this study**

**Table S1 Strains and plasmids used in this study**

| Strains and plasmids | Description | Reference or source |
| --- | --- | --- |
| Strains |  |  |
| JM109 | *E. coli*, cloning strain | Our laboratory |
| *A. succinogenes* CGMCC1593 | *A. succinogenes*, wild type | Our laboratory |
| *ΔAsuc_0914* | *A. succinogenes* derivative, contains *Asuc_0914* knockout | This study |
| *ΔAsuc_1034* | *A. succinogenes* derivative, contains *Asuc_1034* knockout | This study |
| *ΔAsuc_0023* | *A. succinogenes* derivative, contains *Asuc_0023* knockout | This study |
| *ΔAsuc_0715* | *A. succinogenes* derivative, contains *Asuc_0715* knockout | This study |
| *ΔAsuc_0716* | *A. succinogenes* derivative, contains *Asuc_0716* knockout | This study |
| *ΔAsuc_0750* | *A. succinogenes* derivative, contains *Asuc_0750* knockout | This study |
| *ΔAsuc_2056* | *A. succinogenes* derivative, contains *Asuc_2056* knockout | This study |
| Plasmids |  |  |
| pLGZ-dcas9opt-frd-sgRNA | pLGZ922 derivative, contains d*cas9*opt and sgRNA scaffold under the promoter *frd* | Reference 37 |
| pLGZ-dcpf1-917A-frd-crRNA | pLGZ922 derivative, contains d*cpf1* of D917A mutation and crRNA under the promoter *frd* | Reference 37 |
| pLGZ-dcpf1-917A-1006A-frd-crRNA | pLGZ922 derivative, contains d*cpf1* of D917A and E1006A mutation and crRNA under the promoter *frd* | Reference 37 |
| pKCcas9dO | contains *Rep* fragment | Reference 37 |
| rAPOBEC1-nCas9(D10A) | pLGZ-dcas9opt-frd-sgRNA derivative, contains fusion of rAPOBEC1 and nCas9(D10A) | This study |
| rAPOBEC1- dcpf1(D917A) | pLGZ-dcpf1-917A-frd-crRNA derivative, contains fusion of rAPOBEC1 and dcpf1(D917A) | This study |
| rAPOBEC1-dCpf1(D917A-E1006A) | pLGZ-dcpf1-917A-1006A-frd-crRNA derivative, contains fusion of rAPOBEC1 and dcpf1(D917A-E1006A) | This study |
| rAPOBEC1-nCas9(D10A)-UGI | pLGZ-dcas9opt-frd-sgRNA derivative, contains fusion of rAPOBEC1 and nCas9(D10A) and UGI | This study |
| rAPOBEC1- dcpf1(D917A)-UGI | pLGZ-dcpf1-917A-frd-crRNA derivative, contains fusion of rAPOBEC1, dcpf1(D917A) and UGI | This study |
| rAPOBEC1-dCpf1(D917A-E1006A)-UGI | pLGZ-dcpf1-917A-1006A-frd-crRNA derivative, contains fusion of rAPOBEC1, dcpf1(D917A) and UGI | This study |
| nCas9(D10A)max | pLGZ-dcas9opt-frd-sgRNA derivative, contains fusion of rAPOBEC1 and nCas9(D10A) and two copies of UGI | This study |
| dCpf1(D917A)max | pLGZ-dcpf1-917A-frd-crRNA derivative, contains fusion of rAPOBEC1, dcpf1(D917A) and two copies of UGI | This study |
| dCpf1(D917A-E1006A)max | pLGZ-dcpf1-917A-1006A-frd-crRNA derivative, contains fusion of rAPOBEC1, dCpf1(D917A-E1006A) and two copies of UGI | This study |
| **nCas9(D10A)-ABE** | pLGZ-dcas9opt-frd-sgRNA derivative, contains fusion of TadA8e and nCas9(D10A) | This study |
| dCpf1(D917A)-ABE | pLGZ-dcpf1-917A-frd-crRNA derivative, contains fusion of TadA8e and dcpf1(D917A) | This study |
| dCpf1(D917A-E1006A)-ABE | pLGZ-dcpf1-917A-1006A-frd-crRNA derivative, contains fusion of TadA8e and dcpf1(D917A-E1006A) | This study |
| TadA8e-N46L-nCas9(D10A) (**Td-GABE**) | nCas9(D10A)-ABE derivative, contains TadA8e variant with an N46L mutation | This study |
| TadA8e-N46L-nCas9(D10A)-UGI (**Td-CBE**) | Td-GABE derivative, contains TadA8e variant, nCas9(D10A) and UGI | This study |
| TadA8e-N46L-nCas9(D10A)-2UGI (**Td-CBEmax**) | Td-GABE derivative, contains TadA8e variant, nCas9(D10A) and two copies of UGI | This study |
| 1398-CBEmax-1 | nCas9(D10A)max derivative, contains gRNA expression cassette targeting *Asuc_1398* | This study |
| 1398-CBEmax-2 | nCas9(D10A)max derivative, contains gRNA expression cassette targeting *Asuc_1398* | This study |
| 1398-CBEmax-3 | nCas9(D10A)max derivative, contains gRNA expression cassette targeting *Asuc_1398* | This study |
| 1398-CBEmax-4 | nCas9(D10A)max derivative, contains gRNA expression cassette targeting *Asuc_1398* | This study |
| 0715-CBEmax | nCas9(D10A)max derivative, contains gRNA expression cassette targeting *Asuc_0715* | This study |
| 0716-CBEmax | nCas9(D10A)max derivative, contains gRNA expression cassette targeting *Asuc_0716* | This study |
| 0914-CBEmax | nCas9(D10A)max derivative, contains gRNA expression cassette targeting *Asuc_0914* | This study |
| 0301-CBEmax | nCas9(D10A)max derivative, contains gRNA expression cassette targeting *Asuc_0301* | This study |
| 1034-CBEmax | nCas9(D10A)max derivative, contains gRNA expression cassette targeting *Asuc_1034* | This study |
| 1398-ABE-1 | nCas9(D10A)max-ABE derivative, contains gRNA expression cassette targeting *Asuc_1398* | This study |
| 1398-ABE-2 | nCas9(D10A)max-ABE derivative, contains gRNA expression cassette targeting *Asuc_1398* | This study |
| 1398-ABE-3 | nCas9(D10A)max-ABE derivative, contains gRNA expression cassette targeting *Asuc_1398* | This study |
| 1034-ABE-1 | nCas9(D10A)max-ABE derivative, contains gRNA expression cassette targeting *Asuc_1034* | This study |
| 1034-ABE-2 | nCas9(D10A)max-ABE derivative, contains gRNA expression cassette targeting two sites of *Asuc_1034* | This study |
| 1034-ABE-3 | nCas9(D10A)max-ABE derivative, contains gRNA expression cassette targeting three sites of *Asuc_1034* | This study |
| 1034-ABE-4 | nCas9(D10A)max-ABE derivative, contains gRNA expression cassette targeting four sites of *Asuc_1034* | This study |
| 1034-ABE-5 | nCas9(D10A)max-ABE derivative, contains gRNA expression cassette targeting five sites of *Asuc_1034* | This study |
| 1034-ABE-6 | nCas9(D10A)max-ABE derivative, contains gRNA expression cassette targeting six sites of *Asuc_1034* | This study |
| TG-GABE | Td-GABE derivative, contains gRNA expression cassette targeting *Asuc_1575* | This study |
| AG-GABE | Td-GABE derivative, contains gRNA expression cassette targeting *Asuc_1575* | This study |
| CG-GABE | Td-GABE derivative, contains gRNA expression cassette targeting *Asuc_1575* | This study |
| GG-GABE | Td-GABE derivative, contains gRNA expression cassette targeting *Asuc_1575* | This study |
| 1575-Td-CBE-1 | Td-CBE derivative, contains gRNA expression cassette targeting *Asuc_1575* | This study |
| 1575-Td-CBE-2 | Td-CBE derivative, contains gRNA expression cassette targeting two sites of *Asuc_1575* | This study |
| 1034-Td-CBE-1 | Td-CBE derivative, contains gRNA expression cassette targeting *Asuc_1034* | This study |
| 1034-Td-CBE-2 | Td-CBE derivative, contains gRNA expression cassette targeting *Asuc_1034* | This study |
| 0302-Td-CBE-1 | Td-CBE derivative, contains gRNA expression cassette targeting *Asuc_0302* | This study |
| 0302-Td-CBE-2 | Td-CBE derivative, contains gRNA expression cassette targeting *Asuc_0302* | This study |
| 0914-Td-CBE | Td-CBE derivative, contains gRNA expression cassette targeting *Asuc_0914* | This study |
| 1034-Td-CBE | Td-CBE derivative, contains gRNA expression cassette targeting *Asuc_1034* | This study |
| 0301-Td-CBE | Td-CBE derivative, contains gRNA expression cassette targeting *Asuc_0301* | This study |
| 0715-Td-CBE | Td-CBE derivative, contains gRNA expression cassette targeting *Asuc_0715* | This study |
| 0716-Td-CBE | Td-CBE derivative, contains gRNA expression cassette targeting *Asuc_0716* | This study |
| 0023-Td-CBE | Td-CBE derivative, contains gRNA expression cassette targeting *Asuc_0023* | This study |
| 0750-Td-CBE | Td-CBE derivative, contains gRNA expression cassette targeting *Asuc_0750* | This study |
| 2056-Td-CBE | Td-CBE derivative, contains gRNA expression cassette targeting *Asuc_2056* | This study |

**Table S2 The primer sequences used in this study**

| Primer | Sequence (5’ to 3’) |
| --- | --- |
| plgz-R | CACTTTACGTTTCTTTTTCGGTCTAGATCACCTCATTGATA |
| Cas9-16aa-F | TACCTCCGAATCCGCGACCCCGGAATCCGATAAAAAATATTCCATCGG |
| Cpf1-16aa-F | TACCTCCGAATCCGCGACCCCGGAATCCatgtcgatctaccaagagt |
| APOBEC1-16aa-F | CGAAAAAGAAACGTAAAGTGATGTCCTCCGAAACCGGTCC |
| APOBEC1-16aa-R | GGGTCGCGGATTCGGAGGTACCCGGGGTTTCGGAACCGGATTTTAAACCGGTCGCCCATA |
| Cas9-32aa-F | CCCCGGGTACCTCCGAATCCGCGACCCCGGAAAGCTCCGGTGGTTCCTCCGGTGGCAGCGATAAAAAATATTCCATCGG |
| Cpf1-32aa-F | CCCCGGGTACCTCCGAATCCGCGACCCCGGAAAGCTCCGGTGGTTCCTCCGGTGGCAGCtcgatctaccaagagttcgt |
| APOBEC1-32aa-F | GGATTCGGAGGTACCCGGGGTTTCGCTGCCGGAGGAACCACCGGAGGAGCCACCGCTTTTTAAACCGGTCGCCCATA |
| UGI-9aa-F | GGTGGTTCCGGTGGTAGCACCAACTTATCCGATATCATC |
| UGI-9aa-R | GTGCTACCACCGGAACCACCGCTACCACCGGAAACTTTAC |
| UGI2-R | TACCACCGGATAACATTTTGATTTTGTTTTC |
| pLGZ-UGI2-F | CAAAATGTTATCCGGTGGTAGCCCGAAAA |
| pLGZ-UGI2-R | GTGCTACCACCGGAACCACCGCTACCACCGGATAACATTTTGATTTTGTTTTC |
| ABE8e-F | GTGATCTAGAATGAAACGCACGGCCGATGG |
| ABE8e-R | agatcgacatGCTGCCACCGGAGGAACCACCGGAGCTTT |
| Cpf1-32aa-F | CGGTGGCAGCatgtcgatctaccaagagtt |
| cas9-32aa-F | CGGTGGCAGCGATAAAAAATATTCCATCGG |
| pLGZ-ABE8e-R | TGCGTTTCATTCTAGATCACCTCATTGATA |
| Anc689-F | CGAAAAAGAAACGTAAAGTGATGTCCTCCGAAACCGGTC |
| Anc689-R | AGCCACCGCTTTTTAAACCGGTCGCCCAT |
| Rep-F | GACGTTATAACCCAGAAATGGCACGAGCCC |
| Rep-R | TCTAGGGCATCTACGCGACCGCTGTGTCGA |
| pLGZ-Rep-F | GGTCGCGTAGATGCCCTAGAACGTGCAGGTT |
| pLGZ-Rep-R | CATTTCTGGGTTATAACGTCCTGTCGGTCG |
| TadA8e-N46L-F | AGGTTGGTTACGTGCGATCGGTTTACATGA |
| TadA8e-N46L-R | CGATCGCACGTAACCAACCTTCACCGATCACAC |
| pLGZ-P2A-F | ACGTAAAGTGtaaGAGCTCcgtttaggtttacgtcgctaa |
| pLGZ-P2A-R | TCCTCCACGTCTCCAGCCTGCTTCAGCAGGCTGAAGTTAGTAGCTCCGCTTCCAACTTTACGTTTCTTTTTAG |
| P2A-UGI-F | CAGGCTGGAGACGTGGAGGAGAACCCTGGACCTACCAACTTATCCGATATCATC |
| 1398-F1 | ctttgcgaccaatcagtcgcGTTTTAGAGCTAGAAATAGCAAG |
| 1398-R1 | gcgactgattggtcgcaaaggatatttttctccaataaaaagt |
| 1398-F2 | cgacagcgtacagaatttacGTTTTAGAGCTAGAAATAGCAAG |
| 1398-R2 | gtaaattctgtacgctgtcggatatttttctccaataaaaagt |
| 1398-F3 | ccatccaactggcaaactcaGTTTTAGAGCTAGAAATAGCAAG |
| 1398-R3 | tgagtttgccagttggatgggatatttttctccaataaaaagt |
| 1398-F4 | ctaatccctgcgctcattatGTTTTAGAGCTAGAAATAGCAAG |
| 1398-R4 | ataatgagcgcagggattaggatatttttctccaataaaaagt |
| 0715-F1 | acagatttcacaacgttattGTTTTAGAGCTAGAAATAGCAAG |
| 0715-R1 | aataacgttgtgaaatctgtgatatttttctccaataaaaagt |
| 0914-F1 | cgacaggttattgataaactGTTTTAGAGCTAGAAATAGCAAG |
| 0914-R1 | agtttatcaataacctgtcggatatttttctccaataaaaagt |
| 0301-F1 | ccagcttgaaaccatgtcaaGTTTTAGAGCTAGAAATAGCAAG |
| 0301-R1 | ttgacatggtttcaagctgggatatttttctccaataaaaagt |
| 0292-F1 | atgtttttttccttcttttaGTTTTAGAGCTAGAAATAGCAAG |
| 0292-R1 | taaaagaaggaaaaaaacatgatatttttctccaataaaaagt |
| 1034-F1 | tatgatcactgaaaaaagtcGTTTTAGAGCTAGAAATAGCAAG |
| 1034-R1 | gacttttttcagtgatcatagatatttttctccaataaaaagt |
| 0716-F1 | ttacggagaacaatgatgacGTTTTAGAGCTAGAAATAGCAAG |
| 0716-R1 | gtcatcattgttctccgtaagatatttttctccaataaaaagt |
| pLGZ-1034-F2 | CAGCTGGCGTAATAGCGAAGAGGCCCGCAC |
| pLGZ-1034-R2 | ttgtattaaagcggttaaaaGCACCGACTCGGTGCCACTTTTT |
| 1034-F2 | tatgatcactgaaaaaagtcGTTTTAGAGCTAGAAATAGCAAG |
| 1034-R2 | CTTCGCTATTACGCCAGCTG |
| pLGZ-1034-F3 | ttgtattaaagcggttaaaa |
| pLGZ-1034-R3 | TTTTAACCGCTTTAATACAAgatatttttctccaataaaaagt |
| 1034-F3 | aaaaccatggcgaaaatttaGTTTTAGAGCTAGAAATAGCAAG |
| 1034-R3 | ttttaaccgctttaatacaaGCACCGACTCGGTGCCACTTTTT |
| pLGZ-1034-F4 | atgacaacaattttagcgct |
| pLGZ-1034-R4 | taaattttcgccatggttttgatatttttctccaataaaaagt |
| 1034-F4 | aaaaccatggcgaaaatttaGTTTTAGAGCTAGAAATAGCAAG |
| 1034-R4 | agcgctaaaattgttgtcatGCACCGACTCGGTGCCACTTTTT |
| pLGZ-1034-F5 | aaaaccatggcgaaaattta |
| pLGZ-1034-R5 | gaaaagcaaaccgaaccccagatatttttctccaataaaaagt |
| 1034-F5 | tggggttcggtttgcttttcGTTTTAGAGCTAGAAATAGCAAG |
| 1034-R5 | taaattttcgccatggttttGCACCGACTCGGTGCCACTTTTT |
| pLGZ-1034-F6 | tggggttcggtttgcttttc |
| pLGZ-1034-R6 | catcacccgctactttagcggatatttttctccaataaaaagt |
| 1034-F6 | cgctaaagtagcgggtgatgGTTTTAGAGCTAGAAATAGCAAG |
| 1034-R6 | gaaaagcaaaccgaaccccaGCACCGACTCGGTGCCACTTTTT |
| 1575-C6-Td-F | ccaatctgaaaatgtataagGTTTTAGAGCTAGAAATAGCAAG |
| 1575-C6-Td-R | cttatacattttcagattgggatatttttctccaataaaaagt |
| GG-F | tgggcgccaattttaattttGTTTTAGAGCTAGAAATAGCAAG |
| GG-R | aaaattaaaattggcgcccagatatttttctccaataaaaagt |
| AG-F | tcagctaaaaatcggattacGTTTTAGAGCTAGAAATAGCAAG |
| AG-R | gtaatccgatttttagctgagatatttttctccaataaaaagt |
| CG-F | accgttaatgcttaaagaatGTTTTAGAGCTAGAAATAGCAAG |
| CG-R | attctttaagcattaacggtgatatttttctccaataaaaagt |
| TG-F | aatgtataaggggaatgccgGTTTTAGAGCTAGAAATAGCAAG |
| TG-R | cggcattccccttatacattgatatttttctccaataaaaagt |
| pLGZ-1575-F2 | aaataaattttctttgctgtgatatttttctccaataaaaagt |
| pLGZ-1575-R2 | tgggcgccaattttaattttGTTTTAGAGCTAGAAATAGCAAG |
| 1575-F2 | acagcaaagaaaatttattt |
| 1575-R2 | aaaattaaaattggcgcccaGCACCGACTCGGTGCCACTTTTT |
| 1034-td-sg-F | ggatccgcaaacggcgattcGTTTTAGAGCTAGAAATAGCAAG |
| 1034-td-sg-R | gaatcgccgtttgcggatccgatatttttctccaataaaaagt |
| 0023-td-sg-F | tgctcagtccaaacttaaccGTTTTAGAGCTAGAAATAGCAAG |
| 0023-td-sg-R | ggttaagtttggactgagcagatatttttctccaataaaaagt |
| 0715-td-sg-F | attgtgcaaatcaattcttaGTTTTAGAGCTAGAAATAGCAAG |
| 0715-td-sg-R | taagaattgatttgcacaatgatatttttctccaataaaaagt |
| 0716-td-sg-F | catgcagttgcagcacgggcGTTTTAGAGCTAGAAATAGCAAG |
| 0716-td-sg-R | gcccgtgctgcaactgcatggatatttttctccaataaaaagt |
| 0301-td-sg-F | ctaccgcaagcggcatctatGTTTTAGAGCTAGAAATAGCAAG |
| 0301-td-sg-R | atagatgccgcttgcggtaggatatttttctccaataaaaagt |
| 0302-td-sg-F | ccgttgcaaatgttattgcgGTTTTAGAGCTAGAAATAGCAAG |
| 0302-td-sg-R | cgcaataacatttgcaacgggatatttttctccaataaaaagt |
| 2056-td-sg-F | tatccaatctcaccatacgtGTTTTAGAGCTAGAAATAGCAAG |
| 2056-td-sg-R | acgtatggtgagattggatagatatttttctccaataaaaagt |
| 0750-td-sg-F | ctggcaagtcggcgtattggGTTTTAGAGCTAGAAATAGCAAG |
| 0750-td-sg-R | ccaatacgccgacttgccaggatatttttctccaataaaaagt |
| 1034-td-sg-F2 | cccccaaaagtctgaaaaggGTTTTAGAGCTAGAAATAGCAAG |
| 1034-td-sg-R2 | ccttttcagacttttggggggatatttttctccaataaaaagt |
| 0302-td-sg-F2 | attacaagcagtgaaaaaacGTTTTAGAGCTAGAAATAGCAAG |
| 0302-td-sg-R2 | gttttttcactgcttgtaatgatatttttctccaataaaaagt |
| 0914-cexu-F | atggatttcccgcaaattg |
| 0914-cexu-R | aggcgcattggcgaaagta |
| 1575-cexu-F | atgacaacgggcagtttac |
| 1575-cexu-R | actacggccgataaataga |
| 0023-cexu-F | atgtccgatattgcagtaa |
| 0023-cexu-R | caaatcccgaaaggatacg |
| 0715-cecu-F | gcctgcatgggtattgtact |
| 0715-cecu-R | gacgatagaataacagacga |
| 0716-cexu-F | ggcttgaatatgcaggtga |
| 0716-cexu-R | agtacaatacccatgcagg |
| 0292-cexu-F | tacatgaaatgccggatgt |
| 0292-cexu-R | ccgaattatcgctttcttc |
| 1034-cexu-F | gcgtaagctgagttttctg |
| 1034-cexu-R | cacgatagcccgaatcgcc |
| 0301-cexu-F | accgcaccgatggcgggcaa |
| 0301-cexu-R | acatggtttcaagctggtt |
| 0750-cexu-F | atgccaaataaagtaaatag |
| 0750-cexu-R | gactttcgccgtcgcccggt |
| 2056-cexu-F | tagcccgatgtccgcttatc |
| 2056-cexu-R | ccgacagacaaacggccgtc |

**Table S3 The protospacer sequences used in this study**

| Sequence (5’ to 3’) | PAM | Purpose |
| --- | --- | --- |
| ctttgcgaccaatcagtcgc | CGG | Targeting *Asuc_1398* |
| cgacagcgtacagaatttac | CGG | Targeting *Asuc_1398* |
| ccatccaactggcaaactca | AGG | Targeting *Asuc_1398* |
| ctaatccctgcgctcattat | CGG | Targeting *Asuc_1398* |
| acagatttcacaacgttatt | TGG | Targeting *Asuc_0715* |
| cgacaggttattgataaact | CGG | Targeting *Asuc_0914* |
| ccagcttgaaaccatgtcaa | TGG | Targeting *Asuc_0301* |
| atgtttttttccttctttta | AGG | Targeting *Asuc_0292* |
| tatgatcactgaaaaaagtc | AGG | Targeting *Asuc_1034* |
| ttacggagaacaatgatgac | GGG | Targeting *Asuc_0716* |
| cgctaaagtagcgggtgatg | TGG | Targeting *Asuc_1034* |
| ttgtattaaagcggttaaaa | CGG | Targeting *Asuc_1034* |
| aaaaccatggcgaaaattta | TGG | Targeting *Asuc_1034* |
| atgacaacaattttagcgct | CGG | Targeting *Asuc_1034* |
| tatgatcactgaaaaaagtc | AGG | Targeting *Asuc_1034* |
| ggttgaaccggatttcacct | TGG | Targeting *Asuc_1034* |
| ccaatctgaaaatgtataag | GGG | Targeting *Asuc_1575* |
| tgggcgccaattttaatttt | AGG | Targeting *Asuc_1575* |
| tcagctaaaaatcggattac | GGG | Targeting *Asuc_1575* |
| accgttaatgcttaaagaat | GGG | Targeting *Asuc_1575* |
| aatgtataaggggaatgccg | AGG | Targeting *Asuc_1575* |
| tgggcgccaattttaatttt | AGG | Targeting *Asuc_1575* |
| acagcaaagaaaatttattt | CGG | Targeting *Asuc_1575* |
| ggatccgcaaacggcgattc | GGG | Targeting *Asuc_1034* |
| tgctcagtccaaacttaacc | AGG | Targeting *Asuc_0023* |
| attgtgcaaatcaattctta | CGG | Targeting *Asuc_0715* |
| catgcagttgcagcacgggc | TGG | Targeting *Asuc_0716* |
| ctaccgcaagcggcatctat | CGG | Targeting *Asuc_0301* |
| ccgttgcaaatgttattgcg | CGG | Targeting *Asuc_0302* |
| tatccaatctcaccatacgt | TGG | Targeting *Asuc_2056* |
| ctggcaagtcggcgtattgg | CGG | Targeting *Asuc_0750* |
| cccccaaaagtctgaaaagg | TGG | Targeting *Asuc_1034* |
| attacaagcagtgaaaaaac | AGG | Targeting *Asuc_0302* |


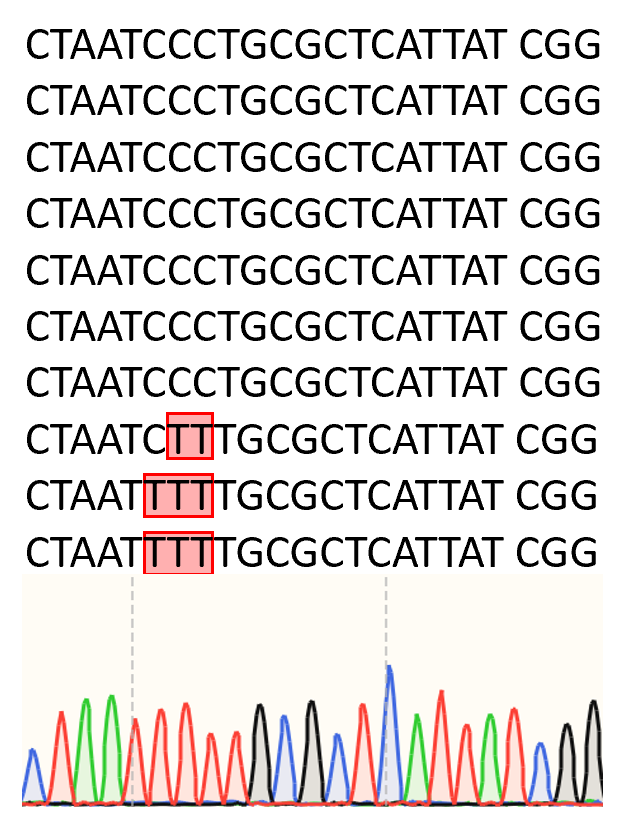


**Fig. S1 The sequencing results of Anc689-nCas9(D10A) targeting *LacZ* gene in *A. succinogenes***


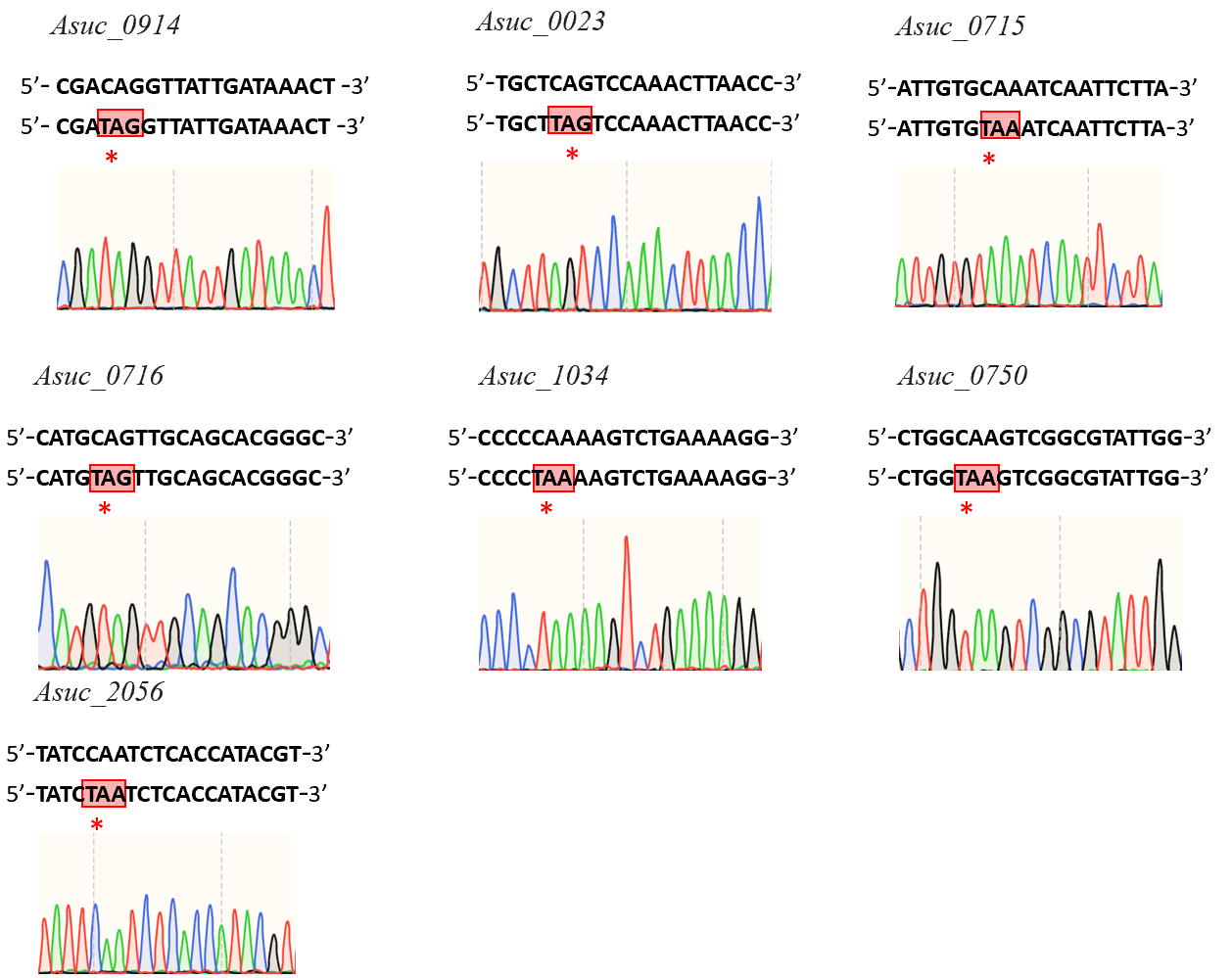
**Fig. S2 The sequencing results of STOP codon introduction targeting genes of transporters in *A. succinogenes***

Supplementary sequences S1 The nCas9(D10A)-CBEmax sequences in this study

CCGAAAAAGAAACGTAAAGTGATGTCCTCCGAAACCGGTCCGGTGGCGGTGGATCCGACCTTACGTCGCCGTATCGAACCGCATGAATTCGAAGTGTTCTTCGATCCGCGTGAATTACGTAAAGAAACCTGTTTATTATATGAAATCAACTGGGGTGGTCGTCATTCCATCTGGCGTCATACCTCCCAAAACACCAACAAACATGTGGAAGTGAACTTCATCGAAAAATTCACCACCGAACGTTATTTCTGTCCGAACACCCGTTGTTCCATCACCTGGTTCTTATCCTGGTCCCCGTGTGGTGAATGTTCCCGTGCGATCACCGAATTCTTATCCCGCTATCCGCATGTGACCTTATTCATCTATATCGCGCGTTTATATCATCATGCGGATCCGCGTAACCGTCAAGGTTTACGTGATTTAATCTCCTCCGGTGTGACCATCCAAATCATGACCGAACAAGAATCCGGTTATTGTTGGCGTAACTTCGTGAACTATTCCCCGTCCAACGAAGCGCATTGGCCGCGTTATCCTCATTTATGGGTGCGTTTATATGTGTTAGAATTATATTGTATCATCTTAGGTTTACCGCCGTGTTTAAACATCTTACGTCGTAAACAACCGCAATTAACCTTCTTCACCATCGCGTTACAATCCTGTCATTATCAACGTTTACCGCCGCATATCTTATGGGCGACCGGTTTAAAAAGCGGTGGCTCCTCCGGTGGTTCCTCCGGCAGCGAAACCCCGGGTACCTCCGAATCCGCGACCCCGGAAAGCTCCGGTGGTTCCTCCGGTGGCAGCGATAAAAAATATTCCATCGGTTTAgcaATCGGTACCAACTCCGTGGGTTGGGCGGTGATCACCGATGATTATAAAGTTCCGAGCAAAAAATTAAAAGGTTTAGGTAACACCGATCGTCATGGTATCAAAAAAAACTTAATCGGTGCGTTATTATTCGATTCCGGTGAAACCGCGGAAGCGACCCGTTTAAAACGTACCGCGCGTCGCCGTTATACCCGTCGTAAAAACCGTATCTGTTATTTACAAGAAATCTTCTCCAATGAAATGGCGAAAGTGGATGATTCCTTCTTCCATCGTTTAGAAGAATCCTTCTTAGTGGAAGAAGATAAAAAACATGAACGTCATCCGATCTTCGGTAACATCGTGGATGAAGTGGCGTATCATGAAAAATATCCGACCATCTATCATTTACGTAAAAAATTAGCGGATTCCACCGATAAAGTGGATTTACGTTTAATCTATTTAGCGTTAGCGCATATGATCAAATTCCGTGGTCATTTCTTAATCGAAGGTGATTTAAACCCGGATAACTCCGATGTGGATAAATTATTCATCCAATTAGTGCAAACCTATAACCAATTATTCGAAGAAAACCCGATCAACGCGTCCCGTGTGGATGCGAAAGCGATCTTATCCGCGCGTTTATCCAAATCCCGTCGTTTAGAGAATTTAATCGCGCAATTACCGGGTGAAAAAAAAAACGGTTTATTCGGTAACTTAATCGCGTTAAGCTTAGGTTTAACCCCGAACTTCAAATCCAACTTCGATTTAGCGGAAGATGCGAAATTACAATTATCCAAAGATACCTATGATGACGATTTAGATAACTTATTAGCGCAAATCGGTGATCAATATGCGGATTTATTCTTAGCGGCGAAAAACTTATCCGACGCGACCTTATTAAGCGATATCTTACGTGTGAACTCCGAAATCACCAAAGCGCCGTTATCCGCGTCCATGATCAAACGTTATGATGAACATCATCAAGATTTAACCTTATTAAAAGCGTTAGTGCGTCAACAATTACCGGAAAAATATAAAGAAATCTTCTTCGACCAATCCAAAAACGGTTATGCGGGTTATATCGATGGTGGTGCGTCCCAAGAAGAATTCTATAAATTCATCAAACCGATCTTAGAAAAGATGGACGGTACCGAAGAATTATTAGCGAAATTAAACCGTGAAGATTTATTACGTAAACAACGTACCTTCGATAACGGTTCCATCCCGTATCAAATCCATTTAGGCGAATTACATGCGATCTTACGTCGTCAAGAAGATTTCTATCCGTTCTTAAAAGATAACCGTGAAAAAATCGAAAAAATCTTAACCTTCCGTATCCCGTATTATGTGGGTCCGTTAGCGCGTGGTAACTCCCGTTTCGCGTGGATGACCCGTAAATCCGAAGAAACCATCACCCCGTGGAACTTCGAAGAAGTGGTGGATAAAGGTGCGTCCGCGCAATCCTTCATCGAACGTATGACCAACTTCGATAAAAACTTACCGAACGAAAAAGTGTTACCGAAACATTCCTTATTATATGAATATTTCACCGTGTATAACGAATTAACCAAAGTGAAATATGTGACCGAAGGTATGCGTAAACCGGCGTTCTTATCCGGTGAACAAAAAAAAGCGATCGTGGATTTATTATTCAAAACCAACCGTAAAGTGACCGTGAAACAATTAAAAGAAGACTATTTCAAAAAAATCGAATGTTTCGATTCCGTGGAAATCTCCGGTGTGGAAGATCGTTTCAACGCGTCCTTAGGTACCTACCACGATTTATTAAAAATCATCAAAGATAAAGATTTCTTAGACAACGAAGAAAACGAAGATATCTTAGAAGATATCGTGTTAACCTTAACCTTATTCGAAGATCGTGAAATGATCGAAGAACGTTTAAAAACCTATGCGCATTTATTCGATGATAAAGTGATGAAACAATTAAAGCGTCGCCGCTATACCGGTTGGGGTCGTTTATCCCGTAAATTAATCAACGGTATCCGTGATAAACAATCCGGTAAAACCATCTTAGATTTCTTAAAATCCGATGGTTTCGCGAACCGTAACTTCATGCAATTAATCCATGATGATTCCTTAACCTTCAAAGAAGATATCCAAAAAGCGCAAGTGTCCGGTCAAGGTGATTCCTTACATGAACATATCGCGAACTTAGCGGGTTCCCCGGCGATCAAAAAAGGTATCTTACAAACCGTGAAAGTGGTGGATGAATTAGTGAAAGTGATGGGTCGTCATAAACCGGAAAACATCGTGATCGAAATGGCGCGTGAAAACCAAACCACCCAAAAAGGTCAAAAAAACTCCCGTGAACGTATGAAACGTATCGAAGAAGGTATCAAAGAATTAGGTTCCGATATCTTAAAAGAATATCCGGTGGAAAACACCCAATTGCAGAATGAAAAATTATATTTATATTATTTACAAAACGGTCGTGATATGTATGTGGATCAAGAATTAGATATCAACCGTTTATCCGATTATGATGTGGATgcaATCGTGCCGCAATCCTTCTTAAAAGACGACTCCATCGATAACAAAGTGTTAACCCGTTCCGATAAAAACCGTGGTAAATCCGATAACGTGCCGTCCGAGGAAGTGGTGAAAAAAATGAAAAACTATTGGCGTCAATTATTAAACGCGAAATTAATCACCCAACGTAAATTCGATAACTTAACCAAAGCGGAACGTGGTGGTTTATCCGAATTGGATAAAGTGGGTTTCATCAAACGTCAATTAGTGGAAACCCGTCAAATCACCAAACATGTGGCGCAAATCTTAGATTCCCGTATGAACACCAAATATGATGAAAACGATAAATTAATCCGTGAAGTGCGTGTGATCACCTTAAAATCCAAATTAGTGTCCGATTTCCGTAAAGATTTCCAATTCTATAAAGTGCGTGAAATCAACAACTATCATCATGCGCATGATGCGTATTTAAACGCGGTGGTGGGTACCGCGTTAATCAAAAAATATCCTAAATTAGAATCCGAATTCGTGTATGGTGACTATAAAGTGTATGATGTGCGTAAAATGATCGCGAAATCCGAACAAGAAATCGGTAAAGCGACCGCGAAATATTTCTTCTATTCCAACATCATGAACTTCTTCAAAACCGAAATCACCTTAGCGAACGGTGAAATCCGTAAACGTCCGTTAATCGAAACCAACGGTGAAACCGGTGAAATCGTGTGGGATAAAGGTCGTGATTTCGCGACCGTGCGTAAGGTGTTATCCATGCCGCAAGTGAACATCGTGAAAAAAACCGAAGTGCAAACCGGTGGTTTCTCCAAAGAATCCATCTTACCGAAACGTAACTCCGATAAATTAATCGCGCGTAAAAAAGATTGGGACCCGAAAAAATATGGTGGTTTCGATTCCCCGACCGTGGCGTATTCCGTGTTAGTGGTGGCGAAAGTTGAAAAAGGTAAATCCAAAAAGTTAAAATCCGTGAAGGAATTATTAGGCATCACCATCATGGAACGTTCCTCCTTCGAAAAAGATCCGATCGATTTCTTAGAAGCGAAAGGTTATAAAGAAGTGCGTAAAGATTTAATCATCAAATTACCGAAATATTCCTTATTCGAATTAGAAAACGGTCGTAAACGTATGTTAGCGTCCGCGGGTGAATTACAAAAAGGTAACGAATTAGCGTTACCGTCCAAATATGTGAACTTCTTATATTTAGCGTCCCATTATGAAAAATTAAAAGGTAGCCCGGAAGATAACGAACAAAAACAATTGTTCGTTGAACAACATAAACATTATTTAGATGAAATCATCGAACAAATCTCCGAATTCTCCAAACGTGTGATCTTAGCGGATGCGAACTTGGACAAAGTGTTATCCGCGTATAACAAACATCGTGACAAACCGATCCGTGAACAAGCGGAAAACATCATCCATTTATTCACCTTAACCAACTTAGGTGCGCCGGCGGCGTTCAAATATTTCGATACCACCATCGATCGTAAACGTTATACCTCCACCAAAGAAGTGTTAGACGCGACCTTAATTCATCAATCCATCACCGGTTTATATGAAACCCGTATCGATTTATCCCAATTAGGTGGTGATTCCGGTGGTAGCAAGCGTACCGCGGATGGCTCCGAGTTTGAACCTAAAAAGAAACGTAAAGTTTCCGGTGGTAGCGGTGGTTCCGGTGGTAGCACCAACTTATCCGATATCATCGAAAAAGAAACCGGTAAACAATTAGTGATCCAAGAATCCATCTTAATGTTACCGGAAGAAGTGGAAGAAGTGATCGGTAACAAACCGGAATCCGATATCTTAGTGCATACCGCGTATGATGAATCCACCGATGAAAACGTGATGTTATTAACCTCCGATGCGCCGGAATATAAACCGTGGGCGTTGGTGATCCAAGATTCCAACGGTGAAAACAAAATCAAAATGTTATCCGGTGGTAGCGGTGGTTCCGGTGGTAGCACCAACTTATCCGATATCATCGAAAAAGAAACCGGTAAACAATTAGTGATCCAAGAATCCATCTTAATGTTACCGGAAGAAGTGGAAGAAGTGATCGGTAACAAACCGGAATCCGATATCTTAGTGCATACCGCGTATGATGAATCCACCGATGAAAACGTGATGTTATTAACCTCCGATGCGCCGGAATATAAACCGTGGGCGTTGGTGATCCAAGATTCCAACGGTGAAAACAAAATCAAAATGTTATCCGGTGGTAGCCCGAAAAAGAAACGTAAAGTGTAA

Supplementary sequences S2 The nCas9(D10A)-ABE plasmid sequences in this study

CCGAAAAAGAAACGTAAAGTGTCCGAAGTGGAATTCTCCCATGAATATTGGATGCGTCATGCGTTAACCTTAGCGAAACGTGCGCGTGATGAACGTGAAGTGCCGGTGGGTGCGGTGTTAGTGTTAAACAACCGTGTGATCGGTGAAGGTTGGAACCGTGCGATCGGTTTACATGATCCGACCGCGCATGCGGAAATCATGGCGTTACGTCAAGGTGGTTTAGTGATGCAAAACTATCGTTTAATCGATGCGACCTTATATGTGACCTTCGAACCGTGTGTGATGTGTGCGGGTGCGATGATCCATTCCCGTATCGGTCGTGTGGTGTTCGGTGTGCGTAACTCCAAACGTGGTGCGGCGGGTTCCTTAATGAACGTGTTAAACTATCCGGGTATGAACCATCGTGTGGAAATCACCGAAGGTATCTTAGCGGATGAATGTGCGGCGTTATTATGTGATTTCTATCGTATGCCGCGTCAAGTGTTCAACGCGCAAAAAAAAGCGCAATCCTCCATCAACAGCGGTGGCTCCTCCGGTGGTTCCTCCGGCAGCGAAACCCCGGGTACCTCCGAATCCGCGACCCCGGAAAGCTCCGGTGGTTCCTCCGGTGGCAGCGATAAAAAATATTCCATCGGTTTAgcaATCGGTACCAACTCCGTGGGTTGGGCGGTGATCACCGATGATTATAAAGTTCCGAGCAAAAAATTAAAAGGTTTAGGTAACACCGATCGTCATGGTATCAAAAAAAACTTAATCGGTGCGTTATTATTCGATTCCGGTGAAACCGCGGAAGCGACCCGTTTAAAACGTACCGCGCGTCGCCGTTATACCCGTCGTAAAAACCGTATCTGTTATTTACAAGAAATCTTCTCCAATGAAATGGCGAAAGTGGATGATTCCTTCTTCCATCGTTTAGAAGAATCCTTCTTAGTGGAAGAAGATAAAAAACATGAACGTCATCCGATCTTCGGTAACATCGTGGATGAAGTGGCGTATCATGAAAAATATCCGACCATCTATCATTTACGTAAAAAATTAGCGGATTCCACCGATAAAGTGGATTTACGTTTAATCTATTTAGCGTTAGCGCATATGATCAAATTCCGTGGTCATTTCTTAATCGAAGGTGATTTAAACCCGGATAACTCCGATGTGGATAAATTATTCATCCAATTAGTGCAAACCTATAACCAATTATTCGAAGAAAACCCGATCAACGCGTCCCGTGTGGATGCGAAAGCGATCTTATCCGCGCGTTTATCCAAATCCCGTCGTTTAGAGAATTTAATCGCGCAATTACCGGGTGAAAAAAAAAACGGTTTATTCGGTAACTTAATCGCGTTAAGCTTAGGTTTAACCCCGAACTTCAAATCCAACTTCGATTTAGCGGAAGATGCGAAATTACAATTATCCAAAGATACCTATGATGACGATTTAGATAACTTATTAGCGCAAATCGGTGATCAATATGCGGATTTATTCTTAGCGGCGAAAAACTTATCCGACGCGACCTTATTAAGCGATATCTTACGTGTGAACTCCGAAATCACCAAAGCGCCGTTATCCGCGTCCATGATCAAACGTTATGATGAACATCATCAAGATTTAACCTTATTAAAAGCGTTAGTGCGTCAACAATTACCGGAAAAATATAAAGAAATCTTCTTCGACCAATCCAAAAACGGTTATGCGGGTTATATCGATGGTGGTGCGTCCCAAGAAGAATTCTATAAATTCATCAAACCGATCTTAGAAAAGATGGACGGTACCGAAGAATTATTAGCGAAATTAAACCGTGAAGATTTATTACGTAAACAACGTACCTTCGATAACGGTTCCATCCCGTATCAAATCCATTTAGGCGAATTACATGCGATCTTACGTCGTCAAGAAGATTTCTATCCGTTCTTAAAAGATAACCGTGAAAAAATCGAAAAAATCTTAACCTTCCGTATCCCGTATTATGTGGGTCCGTTAGCGCGTGGTAACTCCCGTTTCGCGTGGATGACCCGTAAATCCGAAGAAACCATCACCCCGTGGAACTTCGAAGAAGTGGTGGATAAAGGTGCGTCCGCGCAATCCTTCATCGAACGTATGACCAACTTCGATAAAAACTTACCGAACGAAAAAGTGTTACCGAAACATTCCTTATTATATGAATATTTCACCGTGTATAACGAATTAACCAAAGTGAAATATGTGACCGAAGGTATGCGTAAACCGGCGTTCTTATCCGGTGAACAAAAAAAAGCGATCGTGGATTTATTATTCAAAACCAACCGTAAAGTGACCGTGAAACAATTAAAAGAAGACTATTTCAAAAAAATCGAATGTTTCGATTCCGTGGAAATCTCCGGTGTGGAAGATCGTTTCAACGCGTCCTTAGGTACCTACCACGATTTATTAAAAATCATCAAAGATAAAGATTTCTTAGACAACGAAGAAAACGAAGATATCTTAGAAGATATCGTGTTAACCTTAACCTTATTCGAAGATCGTGAAATGATCGAAGAACGTTTAAAAACCTATGCGCATTTATTCGATGATAAAGTGATGAAACAATTAAAGCGTCGCCGCTATACCGGTTGGGGTCGTTTATCCCGTAAATTAATCAACGGTATCCGTGATAAACAATCCGGTAAAACCATCTTAGATTTCTTAAAATCCGATGGTTTCGCGAACCGTAACTTCATGCAATTAATCCATGATGATTCCTTAACCTTCAAAGAAGATATCCAAAAAGCGCAAGTGTCCGGTCAAGGTGATTCCTTACATGAACATATCGCGAACTTAGCGGGTTCCCCGGCGATCAAAAAAGGTATCTTACAAACCGTGAAAGTGGTGGATGAATTAGTGAAAGTGATGGGTCGTCATAAACCGGAAAACATCGTGATCGAAATGGCGCGTGAAAACCAAACCACCCAAAAAGGTCAAAAAAACTCCCGTGAACGTATGAAACGTATCGAAGAAGGTATCAAAGAATTAGGTTCCGATATCTTAAAAGAATATCCGGTGGAAAACACCCAATTGCAGAATGAAAAATTATATTTATATTATTTACAAAACGGTCGTGATATGTATGTGGATCAAGAATTAGATATCAACCGTTTATCCGATTATGATGTGGATgcaATCGTGCCGCAATCCTTCTTAAAAGACGACTCCATCGATAACAAAGTGTTAACCCGTTCCGATAAAAACCGTGGTAAATCCGATAACGTGCCGTCCGAGGAAGTGGTGAAAAAAATGAAAAACTATTGGCGTCAATTATTAAACGCGAAATTAATCACCCAACGTAAATTCGATAACTTAACCAAAGCGGAACGTGGTGGTTTATCCGAATTGGATAAAGTGGGTTTCATCAAACGTCAATTAGTGGAAACCCGTCAAATCACCAAACATGTGGCGCAAATCTTAGATTCCCGTATGAACACCAAATATGATGAAAACGATAAATTAATCCGTGAAGTGCGTGTGATCACCTTAAAATCCAAATTAGTGTCCGATTTCCGTAAAGATTTCCAATTCTATAAAGTGCGTGAAATCAACAACTATCATCATGCGCATGATGCGTATTTAAACGCGGTGGTGGGTACCGCGTTAATCAAAAAATATCCTAAATTAGAATCCGAATTCGTGTATGGTGACTATAAAGTGTATGATGTGCGTAAAATGATCGCGAAATCCGAACAAGAAATCGGTAAAGCGACCGCGAAATATTTCTTCTATTCCAACATCATGAACTTCTTCAAAACCGAAATCACCTTAGCGAACGGTGAAATCCGTAAACGTCCGTTAATCGAAACCAACGGTGAAACCGGTGAAATCGTGTGGGATAAAGGTCGTGATTTCGCGACCGTGCGTAAGGTGTTATCCATGCCGCAAGTGAACATCGTGAAAAAAACCGAAGTGCAAACCGGTGGTTTCTCCAAAGAATCCATCTTACCGAAACGTAACTCCGATAAATTAATCGCGCGTAAAAAAGATTGGGACCCGAAAAAATATGGTGGTTTCGATTCCCCGACCGTGGCGTATTCCGTGTTAGTGGTGGCGAAAGTTGAAAAAGGTAAATCCAAAAAGTTAAAATCCGTGAAGGAATTATTAGGCATCACCATCATGGAACGTTCCTCCTTCGAAAAAGATCCGATCGATTTCTTAGAAGCGAAAGGTTATAAAGAAGTGCGTAAAGATTTAATCATCAAATTACCGAAATATTCCTTATTCGAATTAGAAAACGGTCGTAAACGTATGTTAGCGTCCGCGGGTGAATTACAAAAAGGTAACGAATTAGCGTTACCGTCCAAATATGTGAACTTCTTATATTTAGCGTCCCATTATGAAAAATTAAAAGGTAGCCCGGAAGATAACGAACAAAAACAATTGTTCGTTGAACAACATAAACATTATTTAGATGAAATCATCGAACAAATCTCCGAATTCTCCAAACGTGTGATCTTAGCGGATGCGAACTTGGACAAAGTGTTATCCGCGTATAACAAACATCGTGACAAACCGATCCGTGAACAAGCGGAAAACATCATCCATTTATTCACCTTAACCAACTTAGGTGCGCCGGCGGCGTTCAAATATTTCGATACCACCATCGATCGTAAACGTTATACCTCCACCAAAGAAGTGTTAGACGCGACCTTAATTCATCAATCCATCACCGGTTTATATGAAACCCGTATCGATTTATCCCAATTAGGTGGTGATTCCGGTGGTAGCAAGCGTACCGCGGATGGCTCCGAGTTTGAACCTAAAAAGAAACGTAAAGTTTAA

Supplementary sequences S3 The Td-GABE plasmid sequences in this study

CCGAAAAAGAAACGTAAAGTGTCCGAAGTGGAATTCTCCCATGAATATTGGATGCGTCATGCGTTAACCTTAGCGAAACGTGCGCGTGATGAACGTGAAGTGGCGGTGGGTGCGGTGTTAGTGTTAAACAACCGTGTGATCGGTGAAGGTTGGTTACGTGCGATCGGTTTACATGATCCGACCGCGCATGCGGAAATCATGGCGTTACGTCAAGGTGGTTTAGTGATGCAAAACTATCGTTTAATCGATGCGACCTTATATGTGACCTTCGAACCGTGTGTGATGTGTGCGGGTGCGATGATCCATTCCCGTATCGGTCGTGTGGTGTTCGGTGTGCGTAACTCCAAACGTGGTGCGGCGGGTTCCTTAATGAACGTGTTAAACTATCCGGGTATGAACCATCGTGTGGAAATCACCGAAGGTATCTTAGCGGATGAATGTGCGGCGTTATTATGTGATTTCTATCGTATGCCGCGTCAAGTGTTCAACGCGCAAAAAAAAGCGCAATCCTCCATCAACAGCGGTGGCTCCTCCGGTGGTTCCTCCGGCAGCGAAACCCCGGGTACCTCCGAATCCGCGACCCCGGAAAGCTCCGGTGGTTCCTCCGGTGGCAGCGATAAAAAATATTCCATCGGTTTAgcaATCGGTACCAACTCCGTGGGTTGGGCGGTGATCACCGATGATTATAAAGTTCCGAGCAAAAAATTAAAAGGTTTAGGTAACACCGATCGTCATGGTATCAAAAAAAACTTAATCGGTGCGTTATTATTCGATTCCGGTGAAACCGCGGAAGCGACCCGTTTAAAACGTACCGCGCGTCGCCGTTATACCCGTCGTAAAAACCGTATCTGTTATTTACAAGAAATCTTCTCCAATGAAATGGCGAAAGTGGATGATTCCTTCTTCCATCGTTTAGAAGAATCCTTCTTAGTGGAAGAAGATAAAAAACATGAACGTCATCCGATCTTCGGTAACATCGTGGATGAAGTGGCGTATCATGAAAAATATCCGACCATCTATCATTTACGTAAAAAATTAGCGGATTCCACCGATAAAGTGGATTTACGTTTAATCTATTTAGCGTTAGCGCATATGATCAAATTCCGTGGTCATTTCTTAATCGAAGGTGATTTAAACCCGGATAACTCCGATGTGGATAAATTATTCATCCAATTAGTGCAAACCTATAACCAATTATTCGAAGAAAACCCGATCAACGCGTCCCGTGTGGATGCGAAAGCGATCTTATCCGCGCGTTTATCCAAATCCCGTCGTTTAGAGAATTTAATCGCGCAATTACCGGGTGAAAAAAAAAACGGTTTATTCGGTAACTTAATCGCGTTAAGCTTAGGTTTAACCCCGAACTTCAAATCCAACTTCGATTTAGCGGAAGATGCGAAATTACAATTATCCAAAGATACCTATGATGACGATTTAGATAACTTATTAGCGCAAATCGGTGATCAATATGCGGATTTATTCTTAGCGGCGAAAAACTTATCCGACGCGACCTTATTAAGCGATATCTTACGTGTGAACTCCGAAATCACCAAAGCGCCGTTATCCGCGTCCATGATCAAACGTTATGATGAACATCATCAAGATTTAACCTTATTAAAAGCGTTAGTGCGTCAACAATTACCGGAAAAATATAAAGAAATCTTCTTCGACCAATCCAAAAACGGTTATGCGGGTTATATCGATGGTGGTGCGTCCCAAGAAGAATTCTATAAATTCATCAAACCGATCTTAGAAAAGATGGACGGTACCGAAGAATTATTAGCGAAATTAAACCGTGAAGATTTATTACGTAAACAACGTACCTTCGATAACGGTTCCATCCCGTATCAAATCCATTTAGGCGAATTACATGCGATCTTACGTCGTCAAGAAGATTTCTATCCGTTCTTAAAAGATAACCGTGAAAAAATCGAAAAAATCTTAACCTTCCGTATCCCGTATTATGTGGGTCCGTTAGCGCGTGGTAACTCCCGTTTCGCGTGGATGACCCGTAAATCCGAAGAAACCATCACCCCGTGGAACTTCGAAGAAGTGGTGGATAAAGGTGCGTCCGCGCAATCCTTCATCGAACGTATGACCAACTTCGATAAAAACTTACCGAACGAAAAAGTGTTACCGAAACATTCCTTATTATATGAATATTTCACCGTGTATAACGAATTAACCAAAGTGAAATATGTGACCGAAGGTATGCGTAAACCGGCGTTCTTATCCGGTGAACAAAAAAAAGCGATCGTGGATTTATTATTCAAAACCAACCGTAAAGTGACCGTGAAACAATTAAAAGAAGACTATTTCAAAAAAATCGAATGTTTCGATTCCGTGGAAATCTCCGGTGTGGAAGATCGTTTCAACGCGTCCTTAGGTACCTACCACGATTTATTAAAAATCATCAAAGATAAAGATTTCTTAGACAACGAAGAAAACGAAGATATCTTAGAAGATATCGTGTTAACCTTAACCTTATTCGAAGATCGTGAAATGATCGAAGAACGTTTAAAAACCTATGCGCATTTATTCGATGATAAAGTGATGAAACAATTAAAGCGTCGCCGCTATACCGGTTGGGGTCGTTTATCCCGTAAATTAATCAACGGTATCCGTGATAAACAATCCGGTAAAACCATCTTAGATTTCTTAAAATCCGATGGTTTCGCGAACCGTAACTTCATGCAATTAATCCATGATGATTCCTTAACCTTCAAAGAAGATATCCAAAAAGCGCAAGTGTCCGGTCAAGGTGATTCCTTACATGAACATATCGCGAACTTAGCGGGTTCCCCGGCGATCAAAAAAGGTATCTTACAAACCGTGAAAGTGGTGGATGAATTAGTGAAAGTGATGGGTCGTCATAAACCGGAAAACATCGTGATCGAAATGGCGCGTGAAAACCAAACCACCCAAAAAGGTCAAAAAAACTCCCGTGAACGTATGAAACGTATCGAAGAAGGTATCAAAGAATTAGGTTCCGATATCTTAAAAGAATATCCGGTGGAAAACACCCAATTGCAGAATGAAAAATTATATTTATATTATTTACAAAACGGTCGTGATATGTATGTGGATCAAGAATTAGATATCAACCGTTTATCCGATTATGATGTGGATgcaATCGTGCCGCAATCCTTCTTAAAAGACGACTCCATCGATAACAAAGTGTTAACCCGTTCCGATAAAAACCGTGGTAAATCCGATAACGTGCCGTCCGAGGAAGTGGTGAAAAAAATGAAAAACTATTGGCGTCAATTATTAAACGCGAAATTAATCACCCAACGTAAATTCGATAACTTAACCAAAGCGGAACGTGGTGGTTTATCCGAATTGGATAAAGTGGGTTTCATCAAACGTCAATTAGTGGAAACCCGTCAAATCACCAAACATGTGGCGCAAATCTTAGATTCCCGTATGAACACCAAATATGATGAAAACGATAAATTAATCCGTGAAGTGCGTGTGATCACCTTAAAATCCAAATTAGTGTCCGATTTCCGTAAAGATTTCCAATTCTATAAAGTGCGTGAAATCAACAACTATCATCATGCGCATGATGCGTATTTAAACGCGGTGGTGGGTACCGCGTTAATCAAAAAATATCCTAAATTAGAATCCGAATTCGTGTATGGTGACTATAAAGTGTATGATGTGCGTAAAATGATCGCGAAATCCGAACAAGAAATCGGTAAAGCGACCGCGAAATATTTCTTCTATTCCAACATCATGAACTTCTTCAAAACCGAAATCACCTTAGCGAACGGTGAAATCCGTAAACGTCCGTTAATCGAAACCAACGGTGAAACCGGTGAAATCGTGTGGGATAAAGGTCGTGATTTCGCGACCGTGCGTAAGGTGTTATCCATGCCGCAAGTGAACATCGTGAAAAAAACCGAAGTGCAAACCGGTGGTTTCTCCAAAGAATCCATCTTACCGAAACGTAACTCCGATAAATTAATCGCGCGTAAAAAAGATTGGGACCCGAAAAAATATGGTGGTTTCGATTCCCCGACCGTGGCGTATTCCGTGTTAGTGGTGGCGAAAGTTGAAAAAGGTAAATCCAAAAAGTTAAAATCCGTGAAGGAATTATTAGGCATCACCATCATGGAACGTTCCTCCTTCGAAAAAGATCCGATCGATTTCTTAGAAGCGAAAGGTTATAAAGAAGTGCGTAAAGATTTAATCATCAAATTACCGAAATATTCCTTATTCGAATTAGAAAACGGTCGTAAACGTATGTTAGCGTCCGCGGGTGAATTACAAAAAGGTAACGAATTAGCGTTACCGTCCAAATATGTGAACTTCTTATATTTAGCGTCCCATTATGAAAAATTAAAAGGTAGCCCGGAAGATAACGAACAAAAACAATTGTTCGTTGAACAACATAAACATTATTTAGATGAAATCATCGAACAAATCTCCGAATTCTCCAAACGTGTGATCTTAGCGGATGCGAACTTGGACAAAGTGTTATCCGCGTATAACAAACATCGTGACAAACCGATCCGTGAACAAGCGGAAAACATCATCCATTTATTCACCTTAACCAACTTAGGTGCGCCGGCGGCGTTCAAATATTTCGATACCACCATCGATCGTAAACGTTATACCTCCACCAAAGAAGTGTTAGACGCGACCTTAATTCATCAATCCATCACCGGTTTATATGAAACCCGTATCGATTTATCCCAATTAGGTGGTGATTCCGGTGGTAGCAAGCGTACCGCGGATGGCTCCGAGTTTGAACCTAAAAAGAAACGTAAAGTTTAA

Supplementary sequences S4 The Td-CBE plasmid sequences in this study

CCGAAAAAGAAACGTAAAGTGTCCGAAGTGGAATTCTCCCATGAATATTGGATGCGTCATGCGTTAACCTTAGCGAAACGTGCGCGTGATGAACGTGAAGTGCCGGTGGGTGCGGTGTTAGTGTTAAACAACCGTGTGATCGGTGAAGGTTGGTTACGTGCGATCGGTTTACATGATCCGACCGCGCATGCGGAAATCATGGCGTTACGTCAAGGTGGTTTAGTGATGCAAAACTATCGTTTAATCGATGCGACCTTATATGTGACCTTCGAACCGTGTGTGATGTGTGCGGGTGCGATGATCCATTCCCGTATCGGTCGTGTGGTGTTCGGTGTGCGTAACTCCAAACGTGGTGCGGCGGGTTCCTTAATGAACGTGTTAAACTATCCGGGTATGAACCATCGTGTGGAAATCACCGAAGGTATCTTAGCGGATGAATGTGCGGCGTTATTATGTGATTTCTATCGTATGCCGCGTCAAGTGTTCAACGCGCAAAAAAAAGCGCAATCCTCCATCAACAGCGGTGGCTCCTCCGGTGGTTCCTCCGGCAGCGAAACCCCGGGTACCTCCGAATCCGCGACCCCGGAAAGCTCCGGTGGTTCCTCCGGTGGCAGCGATAAAAAATATTCCATCGGTTTAgcaATCGGTACCAACTCCGTGGGTTGGGCGGTGATCACCGATGATTATAAAGTTCCGAGCAAAAAATTAAAAGGTTTAGGTAACACCGATCGTCATGGTATCAAAAAAAACTTAATCGGTGCGTTATTATTCGATTCCGGTGAAACCGCGGAAGCGACCCGTTTAAAACGTACCGCGCGTCGCCGTTATACCCGTCGTAAAAACCGTATCTGTTATTTACAAGAAATCTTCTCCAATGAAATGGCGAAAGTGGATGATTCCTTCTTCCATCGTTTAGAAGAATCCTTCTTAGTGGAAGAAGATAAAAAACATGAACGTCATCCGATCTTCGGTAACATCGTGGATGAAGTGGCGTATCATGAAAAATATCCGACCATCTATCATTTACGTAAAAAATTAGCGGATTCCACCGATAAAGTGGATTTACGTTTAATCTATTTAGCGTTAGCGCATATGATCAAATTCCGTGGTCATTTCTTAATCGAAGGTGATTTAAACCCGGATAACTCCGATGTGGATAAATTATTCATCCAATTAGTGCAAACCTATAACCAATTATTCGAAGAAAACCCGATCAACGCGTCCCGTGTGGATGCGAAAGCGATCTTATCCGCGCGTTTATCCAAATCCCGTCGTTTAGAGAATTTAATCGCGCAATTACCGGGTGAAAAAAAAAACGGTTTATTCGGTAACTTAATCGCGTTAAGCTTAGGTTTAACCCCGAACTTCAAATCCAACTTCGATTTAGCGGAAGATGCGAAATTACAATTATCCAAAGATACCTATGATGACGATTTAGATAACTTATTAGCGCAAATCGGTGATCAATATGCGGATTTATTCTTAGCGGCGAAAAACTTATCCGACGCGACCTTATTAAGCGATATCTTACGTGTGAACTCCGAAATCACCAAAGCGCCGTTATCCGCGTCCATGATCAAACGTTATGATGAACATCATCAAGATTTAACCTTATTAAAAGCGTTAGTGCGTCAACAATTACCGGAAAAATATAAAGAAATCTTCTTCGACCAATCCAAAAACGGTTATGCGGGTTATATCGATGGTGGTGCGTCCCAAGAAGAATTCTATAAATTCATCAAACCGATCTTAGAAAAGATGGACGGTACCGAAGAATTATTAGCGAAATTAAACCGTGAAGATTTATTACGTAAACAACGTACCTTCGATAACGGTTCCATCCCGTATCAAATCCATTTAGGCGAATTACATGCGATCTTACGTCGTCAAGAAGATTTCTATCCGTTCTTAAAAGATAACCGTGAAAAAATCGAAAAAATCTTAACCTTCCGTATCCCGTATTATGTGGGTCCGTTAGCGCGTGGTAACTCCCGTTTCGCGTGGATGACCCGTAAATCCGAAGAAACCATCACCCCGTGGAACTTCGAAGAAGTGGTGGATAAAGGTGCGTCCGCGCAATCCTTCATCGAACGTATGACCAACTTCGATAAAAACTTACCGAACGAAAAAGTGTTACCGAAACATTCCTTATTATATGAATATTTCACCGTGTATAACGAATTAACCAAAGTGAAATATGTGACCGAAGGTATGCGTAAACCGGCGTTCTTATCCGGTGAACAAAAAAAAGCGATCGTGGATTTATTATTCAAAACCAACCGTAAAGTGACCGTGAAACAATTAAAAGAAGACTATTTCAAAAAAATCGAATGTTTCGATTCCGTGGAAATCTCCGGTGTGGAAGATCGTTTCAACGCGTCCTTAGGTACCTACCACGATTTATTAAAAATCATCAAAGATAAAGATTTCTTAGACAACGAAGAAAACGAAGATATCTTAGAAGATATCGTGTTAACCTTAACCTTATTCGAAGATCGTGAAATGATCGAAGAACGTTTAAAAACCTATGCGCATTTATTCGATGATAAAGTGATGAAACAATTAAAGCGTCGCCGCTATACCGGTTGGGGTCGTTTATCCCGTAAATTAATCAACGGTATCCGTGATAAACAATCCGGTAAAACCATCTTAGATTTCTTAAAATCCGATGGTTTCGCGAACCGTAACTTCATGCAATTAATCCATGATGATTCCTTAACCTTCAAAGAAGATATCCAAAAAGCGCAAGTGTCCGGTCAAGGTGATTCCTTACATGAACATATCGCGAACTTAGCGGGTTCCCCGGCGATCAAAAAAGGTATCTTACAAACCGTGAAAGTGGTGGATGAATTAGTGAAAGTGATGGGTCGTCATAAACCGGAAAACATCGTGATCGAAATGGCGCGTGAAAACCAAACCACCCAAAAAGGTCAAAAAAACTCCCGTGAACGTATGAAACGTATCGAAGAAGGTATCAAAGAATTAGGTTCCGATATCTTAAAAGAATATCCGGTGGAAAACACCCAATTGCAGAATGAAAAATTATATTTATATTATTTACAAAACGGTCGTGATATGTATGTGGATCAAGAATTAGATATCAACCGTTTATCCGATTATGATGTGGATgcaATCGTGCCGCAATCCTTCTTAAAAGACGACTCCATCGATAACAAAGTGTTAACCCGTTCCGATAAAAACCGTGGTAAATCCGATAACGTGCCGTCCGAGGAAGTGGTGAAAAAAATGAAAAACTATTGGCGTCAATTATTAAACGCGAAATTAATCACCCAACGTAAATTCGATAACTTAACCAAAGCGGAACGTGGTGGTTTATCCGAATTGGATAAAGTGGGTTTCATCAAACGTCAATTAGTGGAAACCCGTCAAATCACCAAACATGTGGCGCAAATCTTAGATTCCCGTATGAACACCAAATATGATGAAAACGATAAATTAATCCGTGAAGTGCGTGTGATCACCTTAAAATCCAAATTAGTGTCCGATTTCCGTAAAGATTTCCAATTCTATAAAGTGCGTGAAATCAACAACTATCATCATGCGCATGATGCGTATTTAAACGCGGTGGTGGGTACCGCGTTAATCAAAAAATATCCTAAATTAGAATCCGAATTCGTGTATGGTGACTATAAAGTGTATGATGTGCGTAAAATGATCGCGAAATCCGAACAAGAAATCGGTAAAGCGACCGCGAAATATTTCTTCTATTCCAACATCATGAACTTCTTCAAAACCGAAATCACCTTAGCGAACGGTGAAATCCGTAAACGTCCGTTAATCGAAACCAACGGTGAAACCGGTGAAATCGTGTGGGATAAAGGTCGTGATTTCGCGACCGTGCGTAAGGTGTTATCCATGCCGCAAGTGAACATCGTGAAAAAAACCGAAGTGCAAACCGGTGGTTTCTCCAAAGAATCCATCTTACCGAAACGTAACTCCGATAAATTAATCGCGCGTAAAAAAGATTGGGACCCGAAAAAATATGGTGGTTTCGATTCCCCGACCGTGGCGTATTCCGTGTTAGTGGTGGCGAAAGTTGAAAAAGGTAAATCCAAAAAGTTAAAATCCGTGAAGGAATTATTAGGCATCACCATCATGGAACGTTCCTCCTTCGAAAAAGATCCGATCGATTTCTTAGAAGCGAAAGGTTATAAAGAAGTGCGTAAAGATTTAATCATCAAATTACCGAAATATTCCTTATTCGAATTAGAAAACGGTCGTAAACGTATGTTAGCGTCCGCGGGTGAATTACAAAAAGGTAACGAATTAGCGTTACCGTCCAAATATGTGAACTTCTTATATTTAGCGTCCCATTATGAAAAATTAAAAGGTAGCCCGGAAGATAACGAACAAAAACAATTGTTCGTTGAACAACATAAACATTATTTAGATGAAATCATCGAACAAATCTCCGAATTCTCCAAACGTGTGATCTTAGCGGATGCGAACTTGGACAAAGTGTTATCCGCGTATAACAAACATCGTGACAAACCGATCCGTGAACAAGCGGAAAACATCATCCATTTATTCACCTTAACCAACTTAGGTGCGCCGGCGGCGTTCAAATATTTCGATACCACCATCGATCGTAAACGTTATACCTCCACCAAAGAAGTGTTAGACGCGACCTTAATTCATCAATCCATCACCGGTTTATATGAAACCCGTATCGATTTATCCCAATTAGGTGGTGATTCCGGTGGTAGCAAGCGTACCGCGGATGGCTCCGAGTTTGAACCTAAAAAGAAACGTAAAGTTGGAAGCGGAGCTACTAACTTCAGCCTGCTGAAGCAGGCTGGAGACGTGGAGGAGAACCCTGGACCTACCAACTTATCCGATATCATCGAAAAAGAAACCGGTAAACAATTAGTGATCCAAGAATCCATCTTAATGTTACCGGAAGAAGTGGAAGAAGTGATCGGTAACAAACCGGAATCCGATATCTTAGTGCATACCGCGTATGATGAATCCACCGATGAAAACGTGATGTTATTAACCTCCGATGCGCCGGAATATAAACCGTGGGCGTTGGTGATCCAAGATTCCAACGGTGAAAACAAAATCAAAATGTTATCCGGTGGTAGCCCGAAAAAGAAACGTAAAGTGTAA

1. [↑](#footnote-ref-1)
